# Systematic identification and characterization of circular RNAs involved in flag leaf senescence of rice

**DOI:** 10.1007/s00425-020-03544-6

**Published:** 2021-01-07

**Authors:** Xiaoping Huang, Hongyu Zhang, Rong Guo, Qiang Wang, Xuanzhi Liu, Weigang Kuang, Haiyan Song, Jianglin Liao, Yingjin Huang, Zhaohai Wang

**Affiliations:** Key Laboratory of Crop Physiology, Ecology and Genetic Breeding, Ministry of Education of the P.R. China, Jiangxi Agricultural University, Nanchang, 330045 Jiangxi Province China

**Keywords:** Competing endogenous RNA (ceRNA), Circular RNAs (circRNAs), Leaf senescence, Rice, Weighted gene co-expression network analysis (WGCNA)

## Abstract

**Main conclusion:**

Circular RNAs (circRNAs) identification, expression profiles, and construction of circRNA-parental gene relationships and circRNA-miRNA-mRNA ceRNA networks indicate that circRNAs are involved in flag leaf senescence of rice.

**Abstract:**

Circular RNAs (circRNAs) are a class of 3′-5′ head-to-tail covalently closed non-coding RNAs which have been proved to play important roles in various biological processes. However, no systematic identification of circRNAs associated with leaf senescence in rice has been studied. In this study, a genome-wide high-throughput sequencing analysis was performed using rice flag leaves developing from normal to senescence. Here, a total of 6612 circRNAs were identified, among which, 113 circRNAs were differentially expressed (DE) during the leaf senescence process. Moreover, 4601 (69.59%) circRNAs were derived from the exons or introns of their parental genes, while 2110 (71%) of the parental genes produced only one circRNA. The sequence alignment analysis showed that hundreds of rice circRNAs were conserved among different plant species. Gene Ontology (GO) enrichment analysis revealed that parental genes of DE circRNAs were enriched in many biological processes closely related to leaf senescence. Through weighted gene co-expression network analysis (WGCNA), six continuously down-expressed circRNAs, 18 continuously up-expressed circRNAs and 15 turn-point high-expressed circRNAs were considered to be highly associated with leaf senescence. Additionally, a total of 17 senescence-associated circRNAs were predicted to have parental genes, in which, regulations of three circRNAs to their parental genes were validated by qRT-PCR. The competing endogenous RNA (ceRNA) networks were also constructed. And a total of 11 senescence-associated circRNAs were predicted to act as miRNA sponges to regulate mRNAs, in which, regulation of two circRNAs to eight mRNAs was validated by qRT-PCR. It is discussed that senescence-associated circRNAs were involved in flag leaf senescence probably through mediating their parental genes and ceRNA networks, to participate in several well-studied senescence-associated processes, mainly including the processes of transcription, translation, and posttranslational modification (especially protein glycosylation), oxidation–reduction process, involvement of senescence-associated genes, hormone signaling pathway, proteolysis, and DNA damage repair. This study not only showed the systematic identification of circRNAs involved in leaf senescence of rice, but also laid a foundation for functional research on candidate circRNAs.

**Supplementary Information:**

The online version contains supplementary material available at 10.1007/s00425-020-03544-6.

## Introduction

With rapid development of high-throughput sequencing technology, a large number of noncoding RNAs, such as long noncoding RNAs and microRNAs, were identified and confirmed as the crucial regulator for gene expression and biological function in different biological processes (Li et al. 2015; Shafiq et al. 2016). As a rising star of noncoding RNAs, circular RNAs (circRNAs) are a novel class of endogenous noncoding RNAs characterized by the existence of a covalent bond linking the 3′ and 5′ ends. Different from the traditional linear RNAs terminated with 3′ tails and 5′ caps, circRNAs could form covalently closed ring structures by back-spliced circularization without polyadenylated tails and 5′–3′ polarities (Chen 2016). Therefore, circRNAs are found to be resistant to RNase R, which is an efficient exoribonuclease that degrades mRNAs (Suzuki and Tsukahara 2014). In the past period of time, circRNAs had been perceived as aberrant splicing and potential functions of circRNAs had not been uncovered (Salzman 2016). Although generated mechanism remains largely unclear, it is certain that circRNAs can be classified into exonic, intronic and intergenic circRNAs (Chen 2016).

In recent years, understanding of the circRNAs becomes more and more clear. The majority of circRNAs are found to be expressed at low level in eukaryotes, but some of circRNAs have shown the high expression in a way with spatiotemporal specificity, possibly implying their important roles in these biological processes (Barrett and Salzman 2016). In addition, the sequences of circRNAs are often found to be conserved among various species, indicating the ubiquitous feature of evolutionary conservation of circRNAs (Wang et al. 2014; Zhao et al. 2017). Many conserved circRNAs are derived from important gene loci, which imply their potentially important roles (Meng et al. 2017). Increasing evidences have shown that a large number of circRNAs are identified in animals including human (Chen et al. 2017a), pig (Huang et al. 2018), rat (Wang et al. 2018a, b) and zebrafish (Sharma et al. 2019), demonstrating the important roles of circRNAs in a wide range of biological and developmental processes. The biological functions of circRNAs were poorly revealed in plants, and the first research of plant circRNAs was performed in *Arabidopsis thaliana* (Wang et al. 2014).

Recently, a growing number of circRNAs have been successfully identified in various plant species, which are involved in many biological processes. For example, a total of 3174 circRNAs have been characterized in *Camellia sinensis* from different stage tissues of leaf development, and the integrative analysis of GO and KEGG suggested the candidate role of circRNAs in photosynthetic machinery and metabolites biosynthesis during leaf development (Tong et al. 2018). In *Arabidopsis thaliana*, 168 circRNAs have been identified, in which 41 circRNAs were considered to be differentially expressed in life span, and GO and KEGG analysis showed that the circRNAs might be involved in plant hormone signal transduction, porphyrin and chlorophyll metabolism during leaves senescence (Liu et al. 2017). In sea buckthorn, a total of 2616 circRNAs were identified, in which 252 circRNAs were identified as the differentially expressed circRNAs between three fruit development stages, and the host genes of DE circRNAs are predicted to be involved in carotenoid biosynthesis, lipid synthesis and plant hormone signal transduction during fruit development process (Zhang et al. 2019). Furthermore, 2787 circRNAs were characterized in cucumber, from which circRNAs were found to paly roles in salt stress response by mediating transcription, signal transcription, cell cycle, metabolism adaptation, and ion homeostasis-related pathways (Zhu et al. 2019). A total of 558 potential circRNAs was identified by high-throughput sequencing of an early-flowering trifoliate orange mutant and its wild type during the phase transition stage, in which 176 circRNAs were differentially expressed, possibly playing an important role in the early-flowering process (Zeng et al. 2018). Besides, a total of 705 circRNAs were detected in mature green tomato fruit and red ripening ones, of which 340 circRNAs were differentially expressed to involve in fruit ripening process (Yin et al. 2018). A total of 9994 circRNAs were obtained in young panicles of WXS rice at three development stages, of which 186 circRNAs were significantly differentially expressed to participate in fertility transition process (Wang et al. 2019). Some studies have reported the functional researches of circRNAs in plants. For example, overexpression of Vv-circATS1, a circRNA derived from glycerol-3-P acyltransferase, improved cold tolerance in *Arabidopsis*, while the linear RNA derived from the same sequence was not able to do the same (Gao et al. 2019). Overexpression of another *Arabidopsis* circRNA originated from the first intron of At5g37720 caused pleiotropic phenotypes, including curly and clustered leaves, delayed flowering, and reduced fertility, and altered expression of approximately 800 genes (Cheng et al. 2018). The transgenic rice lines overexpressing circR5g05160 could lead to enhanced disease resistance to *Magnaporthe oryzae* (Fan et al. 2020). Taken together, these results showed the essential functions of circRNAs in various biological processes, but not in leaf senescence of rice.

Additionally, some studies suggested that circRNAs could regulate the expression of their parental genes at the transcriptional and/or posttranscriptional levels (Ye et al. 2015). For example, a transgenic study revealed that overexpression of the rice circRNA circR5g05160 could reduce the expression level of its parental gene *LOC_Os05g05160*, suggesting that the circRNA can act as a negative regulator for its parental gene (Fan et al. 2020). In tomato, overexpression of a circRNA deriving from a key gene in carotenoid biosynthesis (*phytoene synthase 1*) resulted in a decreased abundance of the parental gene *phytoene synthase 1*, and overexpression of a circRNA derived from *phytoene desaturase* showed similar results (Tan et al. 2017). In *Arabidopsis*, a circRNA generated from the sixth exon of the *SEPALLATA 3* gene (*SEP3*) could increase the abundance of the cognate exon 6-skipped alternative splicing variant (*SEP3.3*) by forming R-rings with its own genomic locus. The overexpression of this circRNA led to the formation of flowers with increased petals but fewer stamens (Conn et al. 2017). It has also been reported that circRNAs could act as ceRNA to function as miRNA sponges, and thereby played vital roles in various biological processes. For example, 36 differentially expressed circRNAs were identified in bell pepper, and circRNAs-mediated ceRNAs network demonstrated the functions of circRNAs in chilling response (Zuo et al. 2018). In addition, a total of 61 circRNAs in tomato was found to be potential ceRNAs to combine with miRNAs, and some of the miRNAs had been revealed to participate in the ethylene signaling pathway (Wang et al. 2017a). The ceRNA network has helped a lot to understand the functions of circRNAs in various biological processes.

Leaf senescence is a highly complex developmental process tightly controlled by multiple layers of regulation (Lim et al. 2007; Huang et al. 2019). Leaf senescence may affect plant growth and crop yield (Lim et al. 2007). Rice (*Oryza sativa* L.), a monocotyledonous model organism, is one of the major food crops. Thus, deciphering leaf senescence in rice is of great importance for understanding its molecular regulatory mechanism. In the past years, enough efforts to reveal the molecular mechanisms underlying leaf senescence have been mainly made by transcriptomic (mRNA), proteomic and metabolomic researches (Jeongsik et al. 2016). However, to the best of our knowledge, no systematic identification of circRNAs involved in leaf senescence in rice has been reported.

To identify the circRNAs related to leaf senescence and explore the potential biological functions of circRNAs in rice, a genome-wide high-throughput sequencing was performed using rice flag leaves developing from normal to senescence. A total of 113 differentially expressed (DE) circRNAs were identified and functional enrichment analysis of their parental genes was performed. Subsequently, 39 DE circRNAs were considered to be highly associated with leaf senescence through WGCNA. Furthermore, circRNA-parental gene regulatory relationships and circRNA-miRNA-mRNA ceRNA networks of these senescence-associated DE circRNAs were constructed to decipher their functions on leaf senescence of rice. This study not only showed the systematic identification of circRNAs involving in leaf senescence of rice, but also laid a foundation for functional research on candidate circRNAs.

## Materials and methods

### Plant material and sample collection

Rice cultivar 93-11 (*Oryza sativa* L. subsp. *indica*) was used as the experimental material in this study. The 93-11 seeds were maintained in our laboratory by strict selfing.

The rice 93-11 plants were cultivated in paddy field by normal management way. The flag leaves at booting stage (FL1), flowering stage (FL2), filling stage (FL3), milking maturity stage (FL4) and dough stage (FL5) were collected, respectively. Three biological replicates were used for samples in each stage, with each sample pooled with three flag leaves from three independent plants, flash-frozen in liquid nitrogen, and stored at − 80 °C for subsequent analysis.

### RNA isolation and detection

Total RNAs from each sample at five stages were extracted with TRIzol reagent (Invitrogen, Carlsbad, CA, USA) following the manufacturer’s instructions, respectively. Subsequently, the purity and concentration of total RNAs were detected using NanoDrop 1000 (Thermo Fisher Scientific/NanoDrop Technologies, Wilmington, DE, USA). Then, the integrity of total RNAs was evaluated using the RNA Nano 6000 Assay Kit of the Agilent Bioanalyzer 2100 (Agilent Technologies, Santa Clara, CA, USA). To ensure the application of qualified samples for sequencing, samples with RNA integrity number ≥ 7 were used for subsequent analysis.

### Library construction and RNA sequencing

After quality confirmation of the total RNAs, the library construction for circRNAs was performed according to the protocol as previously described (Tong et al. 2018; Wang et al. 2019). Briefly, a total of 2.0 μg RNA per qualified sample was performed to remove ribosomal RNA using the Ribo-Zero rRNA Removal Kit (Epicentre, Madison, WI, USA). Sequencing libraries were generated using NEBNext^®^ UltraTM Directional RNA Library Prep Kit for Illumina^®^ (NEB, Ipswich, MA, USA) according to manufacturer’s instructions and index codes were added to attribute sequences to each sample. The clustering of the index-coded samples was conducted on a cBot Cluster Generation System using TruSeq PE Cluster Kit v4-cBot-HS (Illumina, San Diego, CA, USA) following the manufacturer’s instructions.

After cluster generation, the resulting libraries were then sequenced on an Illumina Hiseq 2500 platform and pair-end reads were generated. The libraries were constructed and sequenced by Biomarker Biotechnology Corporation (Beijing, China).

### Identification of circRNAs

Prior to genome-wide identification of circRNAs, reads containing adapter, ploy-N and low-quality reads were filtered from the raw reads by in-house Perl scripts. Then, the Q20, Q30, and GC-content were calculated. The resultant clean reads were mapped to the rice reference genome based on MSU-v7.0 (http://rice.plantbiology.msu.edu/) using the HISAT2 software (version 2.0.5). The reads that could not be mapped to the genomes were obtained. For these unmapped reads, the 20-nt anchors were first extracted from both ends and aligned independently to the rice reference genomes to identify the unique anchor positions by a widely using find_circ software (Memczak et al. 2013). The reversed orientation of the aligned anchors suggested circRNA splicing. The anchor alignments were then extended to generate the GT/AG splice sites flanking the complete read alignments and breakpoints. Finally, a candidate circRNA was identified if it had at least two distinct back-spliced reads. The identified circRNAs were output with annotation information. Based on their genomic origins, circRNAs were classified into three types: exonic, intronic, and intergenic circRNAs.

To identify and annotate protein-coding transcripts (mRNAs) from our transcriptome, the clean reads were mapped to the rice reference genome using HISAT2 software (Kim et al. 2015). Next, the mapped reads were assembled using the StringTie software (Pertea et al. 2016). The assembled transcripts were then annotated using the gffcompare program. Finally, the protein-coding transcripts were identified.

### Expression analysis of circRNAs

To compare the expression of circRNAs across five stages in flag leaf of rice, the accounts of back-spliced reads of each circRNA were normalized using the total sequencing reads in a corresponding sample data set (defined as transcripts per million mapped reads, TPMs) as an indicator of their expression levels. To calculate the expression of protein-coding transcripts, the fragments per kilobase of transcript per million mapped reads (FPKMs) value were calculated by StringTie software (1.3.1). And a pairwise differential expression analysis between any two stages (FL1 vs. FL2; FL1 vs. FL3; FL1 vs. FL4; FL1 vs. FL5; FL2 vs. FL3; FL2 vs. FL4; FL2 vs. FL5; FL3 vs. FL4; FL3 vs. FL5; FL4 vs. FL5) was performed using the DESeq R package (1.10.1). In this study, circRNAs with a *P* value < 0.05 and fold change > 1.5 found by DESeq were considered to be differentially expressed whereas DE mRNAs with a false discovery rate < 0.05 and fold change > 2.

To explore whether circRNAs were conserved in different plant species, the back-splicing sequences of all identified circRNAs in this study were compared against the PlantcircBase database with BLASTN (Tong et al. 2018). The detailed method is described as follows. First, we downloaded all the circRNA information in PlantcircBase (http://ibi.zju.edu.cn/plantcircbase/). Then, we analyzed the genomic sequences and back-splicing sequences of circRNAs in the PlantcircBase database to seek for the sequence regulation. Results showed that the genomic sequences linked head with tail to form the complete circRNAs. The back-splicing sequences were produced by the combination of 100 nt at the tail end of the genomics sequences following with 100 nt at the front end of the genomics sequences once the genomics sequences were over 200 nt, thus the junction sites were located in the middle of back-splicing sequences. When the length of genomic sequences was less than 200 nt, the second half of the genomic sequence was moved to the front of the first half to assemble into back-splicing sequences, making the junction sites be located in the middle of back-splicing sequences and the length of back-splicing sequences be equal to those of genomic sequences. Accordingly, we reassembled all identified circRNAs in our study into back-splicing sequences following this strategy. The BLAST analysis (with a threshold of *E* value < 1e-5) was performed to find the conserved circRNAs using the reassembled back-splicing sequences as the query (Tong et al. 2018). Furthermore, circRNAs were defined as spanning the junction sites when the theoretical position of head–tail junction sites was covered by the alignment.

### Gene annotation, GO analysis and KEGG pathway analysis of circRNAs

According to circRNAs alignment to genomic location, the parental genes of the circRNAs were obtained by a Perl script. Databases including GO (Gene Ontology, http://www.geneontology.org/), Pfam (Protein family, http://pfam.xfam.org/), KOG/COG (Clusters of Orthologous Groups of proteins, http://www.ncbi.nlm.nih.gov/KOG), Nr (NCBI nonredundant protein sequences, ftp://ftp.ncbi.nih.gov/blast/db/FASTA/), Swiss-Prot (A manually annotated and reviewed protein sequence database, http://www.uniprot.org/), and KEGG (Kyoto Encyclopedia of Genes and Genomes, http://www.genome.jp/kegg/) were used to perform function annotation of parental genes. GO enrichment analysis on the parental genes of DE circRNAs was carried out using the topGO R packages (version 2.7). Statistical enrichment on the parental genes of DE circRNAs in KEGG biological pathways was performed using KOBAS software (Mao et al. 2005).

### Co-expression network analysis of circRNAs and mRNAs

To further explore the functions of circRNAs related to leaf senescence in rice, WGCNA was conducted using the R package WGCNA (Langfelder and Horvath 2008). First, an unsigned strong co-expression relationship was built based on the adjacency expression matrix of both DE circRNAs and DE mRNAs. Next, the one-step network construction and module detection were adopted using the “dynamic hybrid tree cut algorithm” with the FPKM no less than 0.001, variation of FPKM no less than 0.5, minimum module size of 30, the power value of 5 and merge cut height of 0.217. The other parameters were defined as default values. Highly similar modules were subsequently identified by clustering and then merged into new modules on the basis of eigengenes. The correlation of each module was also analyzed and visualized by the heatmap. Finally, the co-expression network was visualized by Cytoscape software (v.3.5.0).

### Construction of circRNAs–miRNAs–mRNAs network

To explore the coordinated functions of DE circRNAs, the circRNAs–miRNAs–mRNAs ceRNA networks were constructed. First, known mature miRNA sequences for rice were downloaded from miRBase (release 22, October 2018). Subsequently, the identified DE circRNAs and DE mRNAs were used as target prediction library. Then, psRNATarget was used with default parameters to identify miRNA target binding sites (Dai et al. 2018). Regulatory data of miRNAs-DE circRNAs and miRNAs-DE mRNAs were obtained. In addition, the target mRNAs were compared with the DE mRNAs in specific modules to obtain the crossover mRNAs. Based on interaction relationship among DE circRNAs–miRNAs–DE crossover mRNAs, ceRNA networks were constructed and visualized by Cytoscape software (v3.5.0).

### Validation of circRNAs and quantitative real-time PCR (qRT-PCR)

The circRNA candidates were validated using the following procedure. First, the genomic DNA of flag leaf from rice was extracted using the cetyltrimethylammonium bromide method as a negative control for polymerase chain reaction (PCR) with divergent primers. Second, total RNA was isolated using Trizol reagent (Ambion). Then, total RNA solutions without DNA contamination were subjected to reverse transcriptase reactions with the PrimeScript RT Reagent Kit (Takara, Dalian, China) according to manufacturer’s protocol. To validate circRNAs of flag leaf in rice, PCR was performed using divergent and convergent primers. Convergent primers were used as positive controls for linear transcripts, and divergent primers were used to detect the candidate circular template. For each PCR amplification, cDNA or genomic DNA was used with T3 DNA polymerase (Tsingke, Beijing, China), and 36 cycles of PCR were performed to amplify back-spliced junction sites of circRNAs. The PCR products were separated using 1% agarose gel and Sanger sequencing were carried out to further confirm the junction reads by Sangon Biotech Company (Shanghai, China).

Quantitative real-time PCR (RT-qPCR) was performed using SYBR Green PCR kit (TaKaRa) on a CFX 96 Real-Time PCR system (Bio-Rad) to validate the expression levels of circRNAs and protein-coding genes according to the manufacturer’s instructions. Three genes, *UBC* (*LOC_Os02g42314*), *ARF* (*LOC_Os05g41060*) and *Profilin-2* (*LOC_Os06g*05880) were used as internal reference genes to normalize the qRT-PCR data (Wang et al. 2016). The relative RNA expression levels were calculated using the 2^−ΔΔCT^ method (Ren et al. 2018). The reaction was carried out using three biological replicates with three technical replicates. The primers for mRNAs were designed as followed the routine principle, and the divergent primer was designed for qRT-PCR using the ‘out-facing’ strategy to amplify circRNAs. All primers for these genes were designed using the Primer Premier 5 software and showed in Suppl. Table S1.

## Results

### Identification of circRNAs involved in flag leaf senescence of rice

To systematically identify cirRNAs involved in leaf senescence of rice, a total of 15 RNA libraries from rice flag leaves at booting stage (FL1, before flag leaf senescence), flowering stage (FL2, before flag leaf senescence), filling stage (FL3, early stage of flag leaf senescence, or turn-point stage of flag leaf senescence), milking maturity stage (FL4, middle stage of flag leaf senescence) and dough stage (FL5, late stage of flag leaf senescence) were individually constructed, with three biological replicates for each stage (Suppl. Fig. S1). A genome-wide high-throughput RNA sequencing on these 15 libraries was performed using Illumina Hiseq 2500 platform. More than 110 million raw reads were generated from each library, respectively (Table 1). After filtering the adapters and low-quality reads, a total of 288.75 gigabases clean reads were obtained. And clean reads in each library mapped to the *Oryza sativa* reference genome ranged 98.61–99.79% (Table 1). Furthermore, the Q30 of the clean reads was greater than 94.1% and GC contents of the sequencing outputs were 45.67–46.95% (Table 1).

**Table 1:** Summary of circRNAs sequencing in 15 rice flag leaf samples

Sample	Raw reads	Clean_reads	Mapped reads (%)	Uniq Map reads (%)	GC (%)	Q20 (%)	Q30 (%)
FL1-1	123,393,578	61,696,789	99.76	61.58	45.67	98.11	94.29
FL1-2	114,930,654	57,465,327	99.75	62.99	45.85	98.22	94.6
FL1-3	124,927,304	62,463,652	99.79	63.04	46.22	98.22	94.55
FL2-1	131,360,464	65,680,232	99.60	63.37	46	98.22	94.54
FL2-2	130,654,926	65,327,463	99.55	63.31	46.01	98.04	94.1
FL2-3	122,224,704	61,112,352	99.67	63.81	46.77	98.21	94.53
FL3-1	126,829,020	63,414,510	98.90	66.10	46.41	98.22	94.53
FL3-2	159,242,022	79,621,011	99.04	65.29	46.35	98.22	94.5
FL3-3	128,791,336	64,395,668	98.99	66.06	46.52	98.21	94.5
FL4-1	122,445,208	61,222,604	98.71	65.98	46.67	98.41	94.96
FL4-2	143,316,646	71,658,323	98.66	65.96	46.89	98.27	94.64
FL4-3	123,146,180	61,573,090	98.74	65.53	46.37	98.17	94.42
FL5-1	128,511,780	64,255,890	98.73	67.06	46.93	98.1	94.23
FL5-2	125,910,476	62,955,238	98.74	67.18	46.56	98.29	94.65
FL5-3	136,795,808	68,397,904	98.61	66.85	46.95	98.15	94.39

**Fig. 1:**
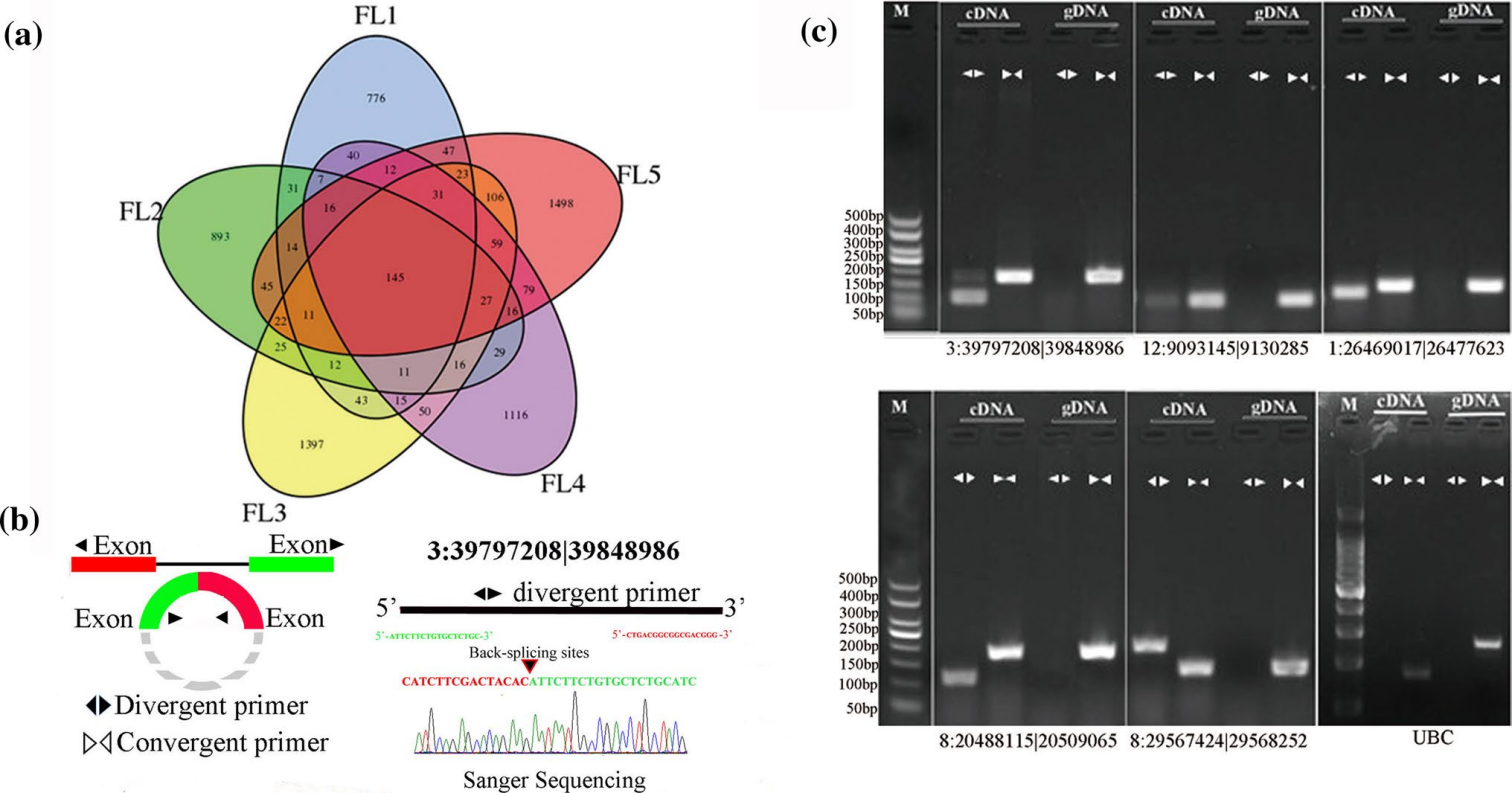
The distribution of circRNAs in different stages and circRNAs validation. **a** A Venn diagram showing the number and distribution of detected circRNAs in five developmental stages. **b** A detailed depiction of circRNA circularization using divergent primers and Sanger sequence validation. **c** PCR amplification results for five predicted circRNAs in genomic DNA and cDNA samples. Divergent primers and convergent primers worked on both cDNA and genomic DNA. UBC as a linear control

After careful screening and further bioinformatic analysis, a total of 6612 circRNAs were identified from 15 flag leaf samples in this study (Suppl. Table S2). Among these identified 6612 circRNAs, 1234, 1320, 1993, 1669, 2151 were, respectively, detected in FL1, FL2, FL3, FL4 and FL5 stage, which had the largest number of identified circRNAs in dough stage (FL5) than others (Fig. 1a). And 145 circRNAs were commonly found in all five stages (Fig. 1a). The result indicated that the expression of circRNAs was specific for the development stage, which is consistent with previous reports that rice circRNAs often expressed specially in different developmental stages (Ye et al. 2015). Besides, circRNAs can be amplified with divergent primers in RNA samples, but not in genomic DNA. In contrast, convergent primers will amplify the linear form of the circRNA templates both in genomic DNA and RNA samples. As a result, the predicted back-splices of five randomly chosen circRNAs were validated by agarose gel electrophoresis and confirmed by Sanger sequencing (Fig. 1b, c).

### Characterization of circRNAs involved in flag leaf senescence of rice

**Fig. 2:**
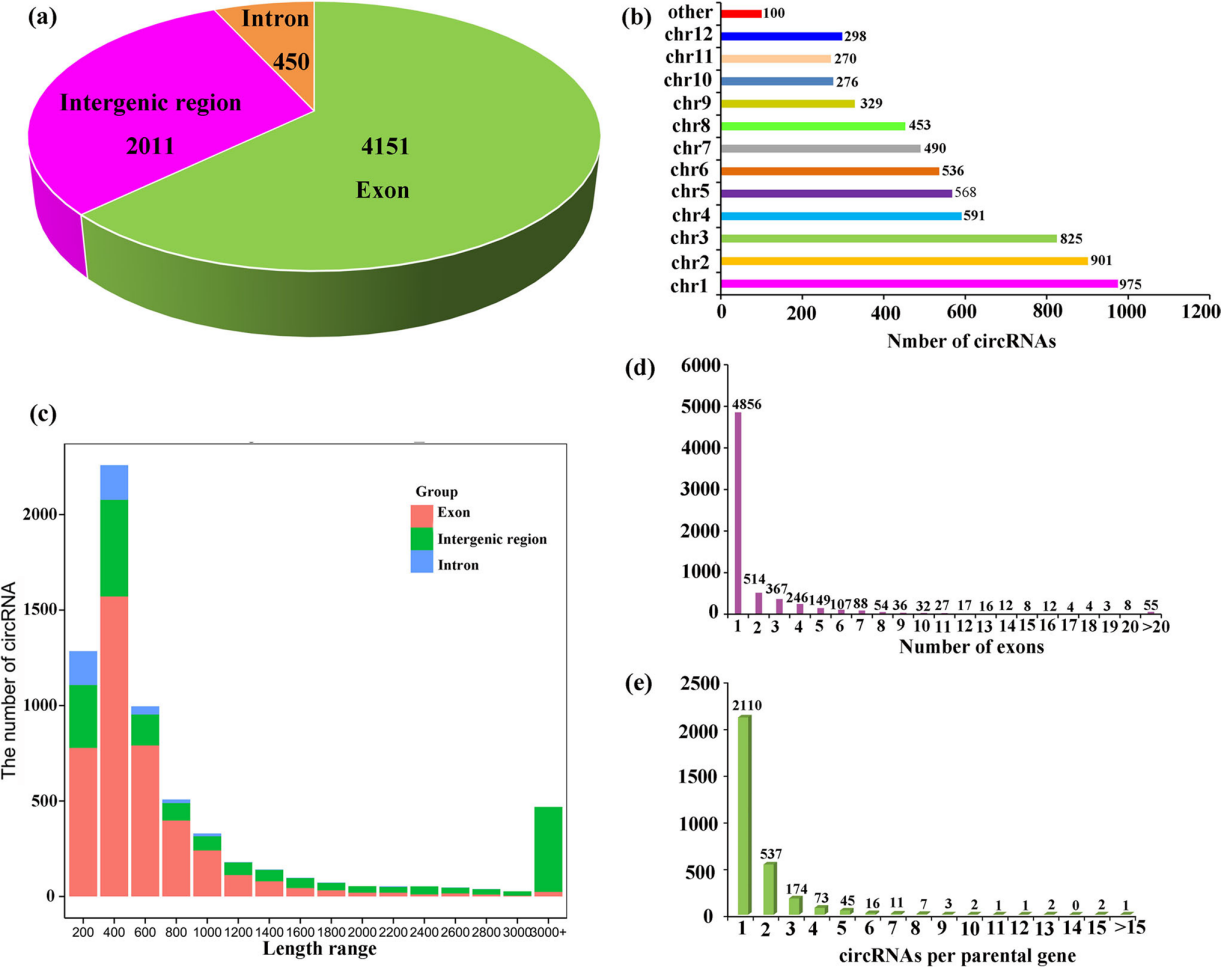
Characterization analysis of circRNAs in rice flag leaves. **a** The number of circRNAs generated from exonic, intronic and intergenic regions. **b** Histogram showed the number of circRNAs detected in each chromosome. **c** Histogram showing the length distribution of circRNAs in flag leaves of rice. **d** Distribution of the number of exons per detected circRNA. **e** Number of detected circRNA per gene

The features of these 6612 identified circRNAs were further analyzed. Genomic origin analysis showed that 4151 (62.78%) of the identified circRNAs were exonic circRNAs, 450 (6.81%) were intronic circRNAs, and that the remaining 2011 (30.41%) were intergenic circRNAs (Fig. 2a, Suppl. Table S2). These results indicated that circRNAs in rice were generated from different genomic regions and mainly from coding regions. The chromosome distribution analysis showed that most of the circRNAs were generated from chromosome 1, followed by chromosomes 2 and 3 (Fig. 2b, Suppl. Table S2). The length count analysis showed that most exonic circRNAs are shorter than 1000 nucleotides (Fig. 2c). Moreover, circular RNAs could possess multiple exons, but 4856 out of 6612 identified circRNAs contained only one annotated exon (Fig. 2d). Annotations results of these circRNAs showed that a total of 4601 circRNAs from identified 6612 circRNAs were derived from 2986 parental genes (Suppl. Table S3), while parental genes of the remaining 2011 circRNAs were not predicted. Interestingly, 2110 (71%) of the parental genes produced only one circRNA, although some parental genes could produce more than one circRNA (Fig. 2e, Suppl. Table S2).

To further explore conservation of circRNAs in different plant species, we did BLAST of the identified 6612 circRNAs against the PlantcircBase database according to the previously described method (Tong et al. 2018). The identified circRNAs were reassembled into back-splicing sequences with the length ranging from 51 to 200 nt (Suppl. Table S4), and the reassembled back-splicing sequences containing the back-splicing junction sites were used for conservation analysis. About 21.3% (1407/6612) of the rice circRNAs were homologous to the circRNA sequence in the database, accounting for homology to approximately 1.1% of the collected plant circRNAs in the database currently (Suppl. Table S4). The identities of circRNAs ranged 78.76–100% and the alignment length ranged from 24 to 200 nt (Suppl. Table S4). In addition, we also identified the circRNAs of which the alignment sequence spanned the junction sites during the BLAST. In this case, we found the alignment sequence of 176 rice circRNAs in our query spanned their own junction sites, while the alignment sequence of 185 rice circRNAs spanned the junction sites of the reference circRNAs in database, and eventually the alignment sequence of 174 rice circRNAs spanned both their own junction sites and those in other 13 plant species (Suppl. Table S4). This result suggested that a number of circRNAs exhibit similar back-splicing patterns across different species, in spite of the type and number of circRNAs may differ in different species.

### Analysis of differentially expressed (DE) circRNAs involved in flag leaf senescence of rice

**Fig. 3:**
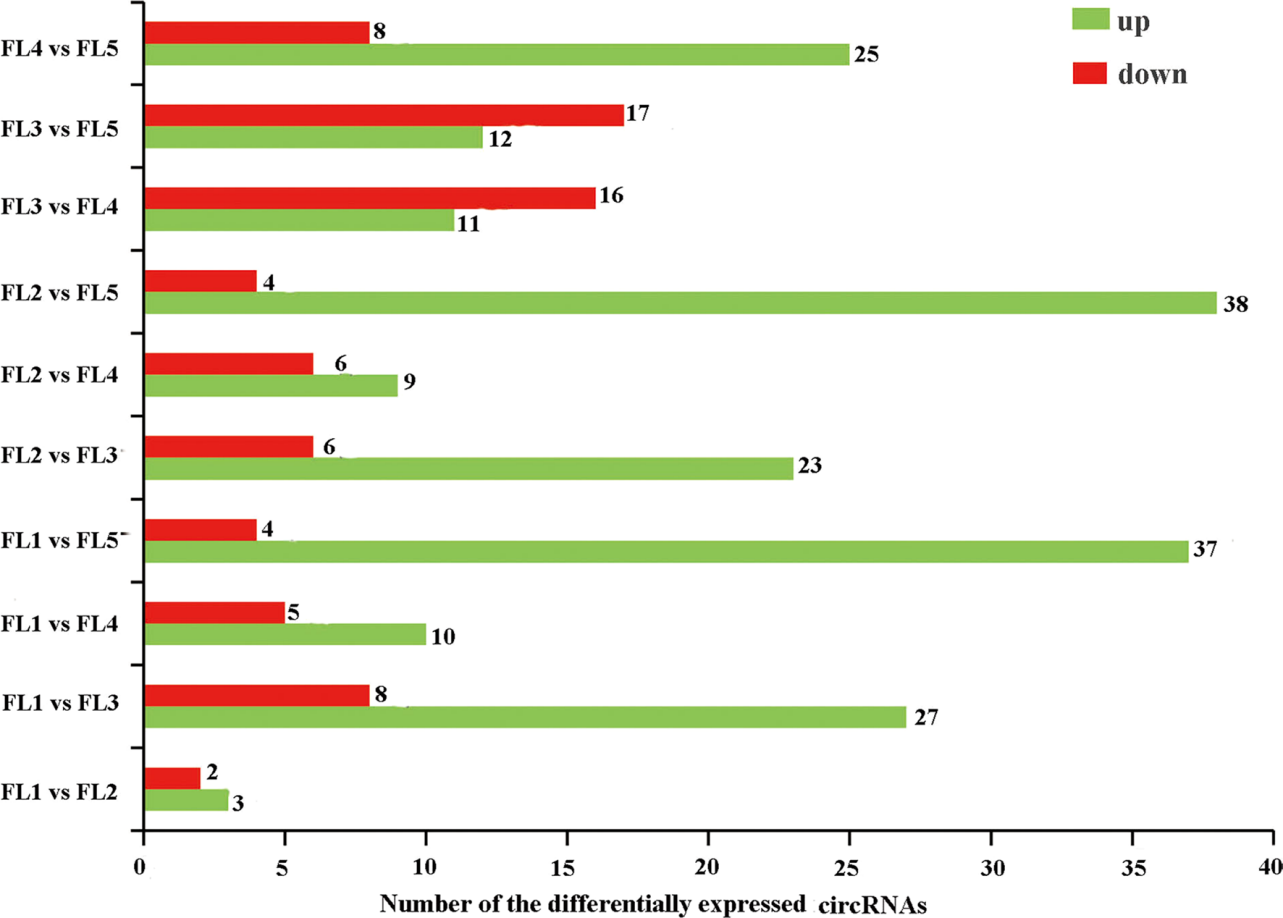
The number of differentially expressed circRNAs in each pairwise comparison group

To reveal the expression patterns of rice circRNAs related to leaf senescence, the 6612 circRNAs identified at five stages were filtered by only retaining those that were detected in least two biological replicates. And the filtered circRNAs were used for the following differential expression analysis. And a total of ten pairwise comparisons groups were analyzed in this study. Based on the screening criteria of fold change > 1.5 and *P* value < 0.05, 5 (FL1 vs. FL2), 35 (FL1 vs. FL3), 15 (FL1 vs. FL4), 41 (FL1 vs. FL5), 29 (FL2 vs. FL3), 15 (FL2 vs. FL4), 42 (FL2 vs. FL5), 27 (FL3 vs. FL4), 29 (FL3 vs. FL5) and 33 (FL4 vs. FL5) DE circRNAs were identified in these ten groups, respectively (Fig. 3, Suppl. Table S5). Eventually, a total of 113 DE circRNAs were obtained (Suppl. Table S5). The number of up- and down-regulated DE circRNAs in each group was also displayed (Fig. 3). For example, four DE circRNAs were up-regulated while 37 DE circRNAs were down-regulated in FL1 vs. FL5 group. Obviously, the number of DE circRNAs in FL1 vs FL2 group was the least, which may imply the little difference of circRNAs between FL1 stage and FL2 stage, which were both before flag leaf senescence. These DE circRNAs identified above may have specific functions in flag leaf senescence of rice.

### Functional enrichment analysis on parental genes of DE circRNAs

**Fig. 4:**
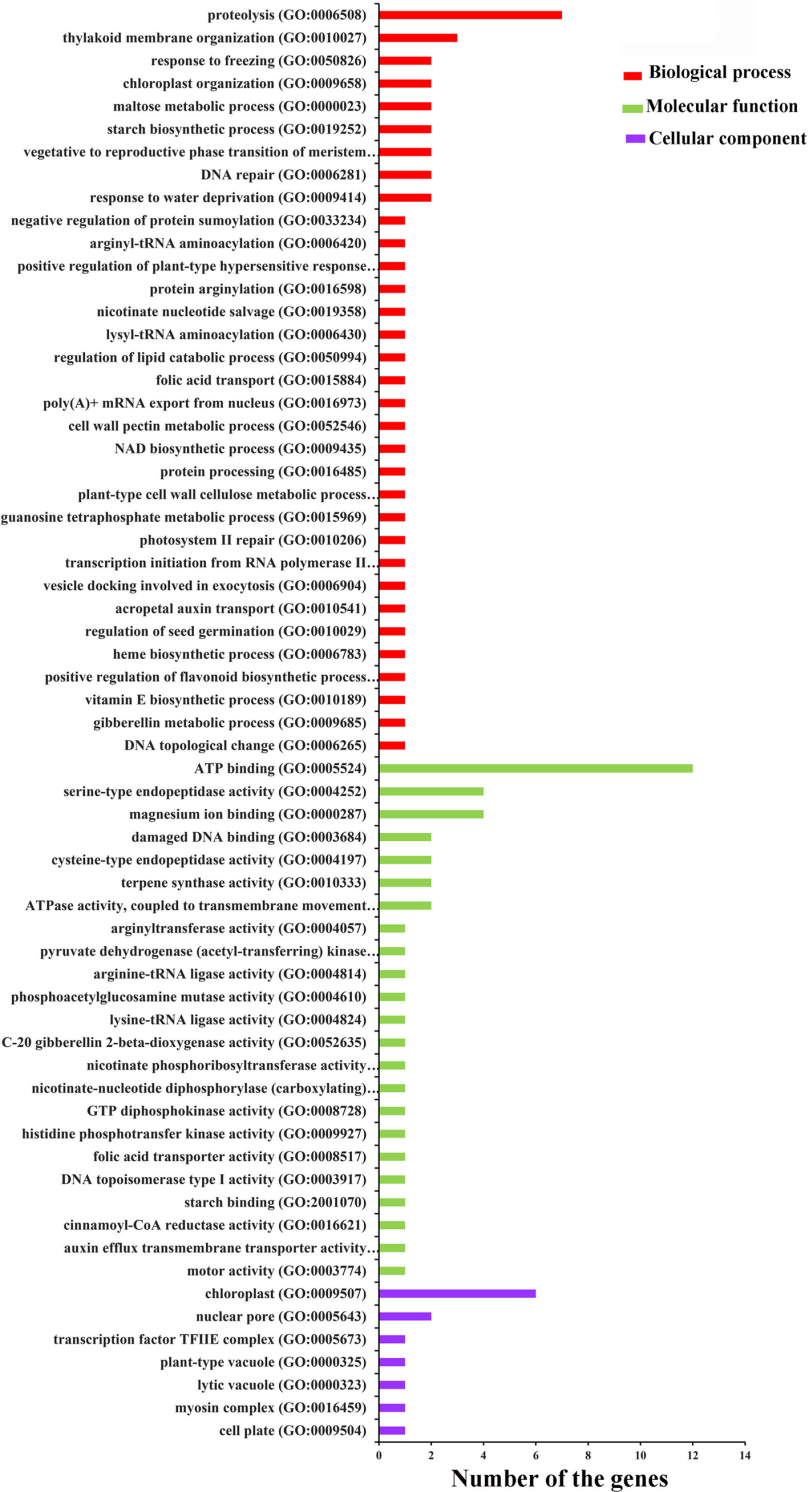
Gene Ontology (GO) enrichment analysis on parental genes of 113 differentially expressed circRNAs

According to circRNAs alignment to genomic location, the parental genes of the 113 identified DE circRNAs were obtained by Perl scripts. At last, a total of 50 parental genes of 51 DE circRNAs were predicted and annotated, whereas the remaining 62 DE circRNAs did not have parental coding genes (Suppl. Table S5). To further investigate the potential functions of the identified DE circRNAs involving in flag leaf senescence, the functional enrichment analysis on these 50 predicted parental genes of 51 DE circRNAs was performed. The Gene Ontology (GO) enrichment analysis showed that these parental genes were assigned to 63 GO terms (*P* < 0.05), among which 7, 23 and 33 GO terms were classified under cellular component, molecular function and biological process, respectively (Fig. 4, Suppl. Table S6). Under cellular component, ‘chloroplast (GO:0,009,507)’ was the most represented GO term, followed second by the category of ‘nuclear pore (GO:0,005,643)’. In the molecular function group, the two main represented categories were ‘ATP binding (GO:0,005,524)’ and ‘serine-type endopeptidase activity (GO:0,004,252)’. Most importantly, many biological processes were found to be closely related to leaf senescence, such as ‘proteolysis (GO:0,006,508)’, ‘thylakoid membrane organization (GO:0,010,027)’, ‘chloroplast organization (GO:0,009,658)’, ‘maltose metabolic process (GO:0,000,023)’, ‘starch biosynthetic process (GO:0,019,252)’, ‘DNA repair (GO:0,006,281)’, ‘nicotinate nucleotide salvage (GO:0,019,358)’, ‘cell wall pectin metabolic process (GO:0,052,546)’, ‘NAD biosynthetic process (GO:0,009,435)’ and ‘plant-type cell wall cellulose metabolic process (GO:0,052,541)’ (Fig. 4). These biological processes referred to proteolysis, carbohydrate metabolism, chloroplast status, DNA damage repair, and oxidation–reduction process.

KEGG pathway analysis was also performed to further explore the functions of the parental genes of DE circRNAs. The result showed that the DE circRNAs-parental genes were significantly enriched in three pathways, including ‘Tropane, piperidine and pyridine alkaloid biosynthesis (ko00960)’, ‘ABC transporters (ko02010)’ and ‘Nicotinate and nicotinamide metabolism (ko00760)’ (Suppl. Table S6). The result may imply that parent genes of circRNAs in flag leaf of rice were involved in oxidation–reduction process and signal transduction.

### Co-expression of circRNAs and mRNAs by WGCNA

**Fig. 5:**
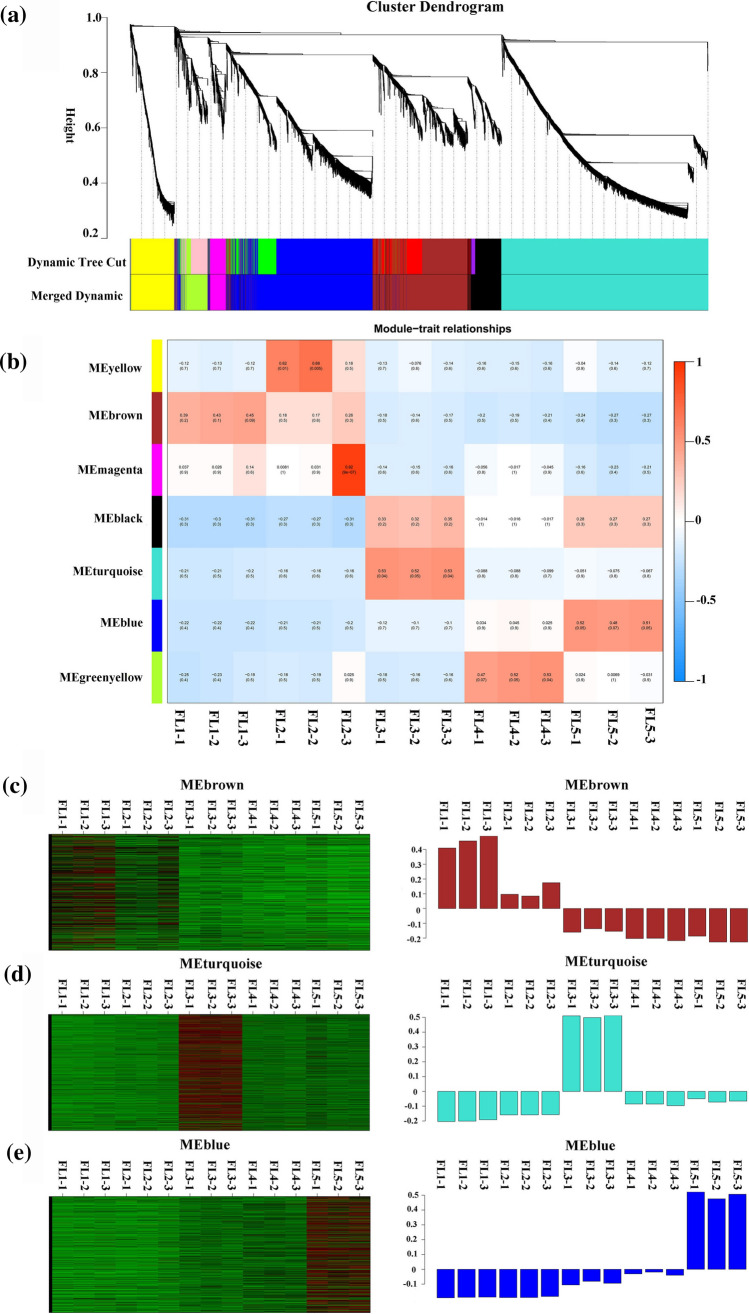
WGCNA of transcripts including differentially expressed (DE) circRNAs and DE mRNAs involved in flag leaf senescence of rice. **a** Hierarchical cluster tree indicating seven modules identified by WGCNA. Each leaf in the tree is one gene and the major tree branches constitute seven modules labeled by different colors. **b** Module–trait correlations and corresponding *P*-values. Each row corresponds to a module and each column corresponds to a specific-stage sample. Each cell at the row–column intersection is color-coded by correlation according to the color legend. **c** Heatmap indicating the eigengene expression profile for the ‘MEbrown’ module. **d** Heatmap indicating the eigengene expression profile for the ‘MEturquoise’ module. **e** Heatmap indicating the eigengene expression profile for the ‘MEblue’ module

To systematically explore the potential regulation functions of circRNAs associated with flag leaf senescence, WGCNA was performed to identify gene sets associated with a specific biological process. First, the protein-coding transcripts were identified by rRNA-depleted library RNA-seq similar to the identification of circRNAs. Then, the DE mRNA transcripts were obtained by pairwise comparison (Suppl. Table S7). Next, the TPM matrix of DE circRNA transcripts and FPKM matrix of DE mRNA transcripts were combined. After bioinformatics processing, a total of seven modules were obtained, in which major tree branches define the modules (labeled with different colors), as shown in the dendrogram (Fig. 5a). The modules closely related to flag leaf senescence were of particular interest to investigate, and the module–trait correlations were displayed in Fig. 5b. Notably, of the seven modules, the ‘MEbrown’ module displayed a continuous down-regulation trend accompanying with the flag leaf development before and after senescence, whereas the ‘MEblue’ module showed a continuous up-regulation trend during this process of leaf senescence (Fig. 5b). In addition, the ‘MEturquoise’ module showed an up-regulation peak at the early-senescence stage, followed by down-regulated expression during leaf senescence, namely termed as the turn-point module of flag leaf senescence (Fig. 5b).

In addition, the heatmaps of ‘MEbrown’ module, ‘MEblue’ module and ‘MEturquoise’ module showed the comprised transcripts which were the most highest expressed in flag leaf at booting stage (FL1, before flag leaf senescence), dough stage (FL5, late stage of flag leaf senescence) and filling stage (FL3, early stage of flag leaf senescence), respectively (Fig. 5c-e). As a result, considering the continuous down-regulation, up-regulation and turn-point trends during the process of flag leaf development from normal to senescence, the ‘MEbrown’, ‘MEblue’ and ‘MEturquoise’ module were considered to be highly associated with flag leaf senescence (Fig. 5). The DE circRNAs clustered in these three modules (‘MEbrown’, ‘MEblue’ and ‘MEturquoise’) were considered to be stage-specific, and senescence-associated circRNAs were further investigated.

### Display of the interested senescence-associated circRNAs and their putative parental genes

**Fig. 6:**
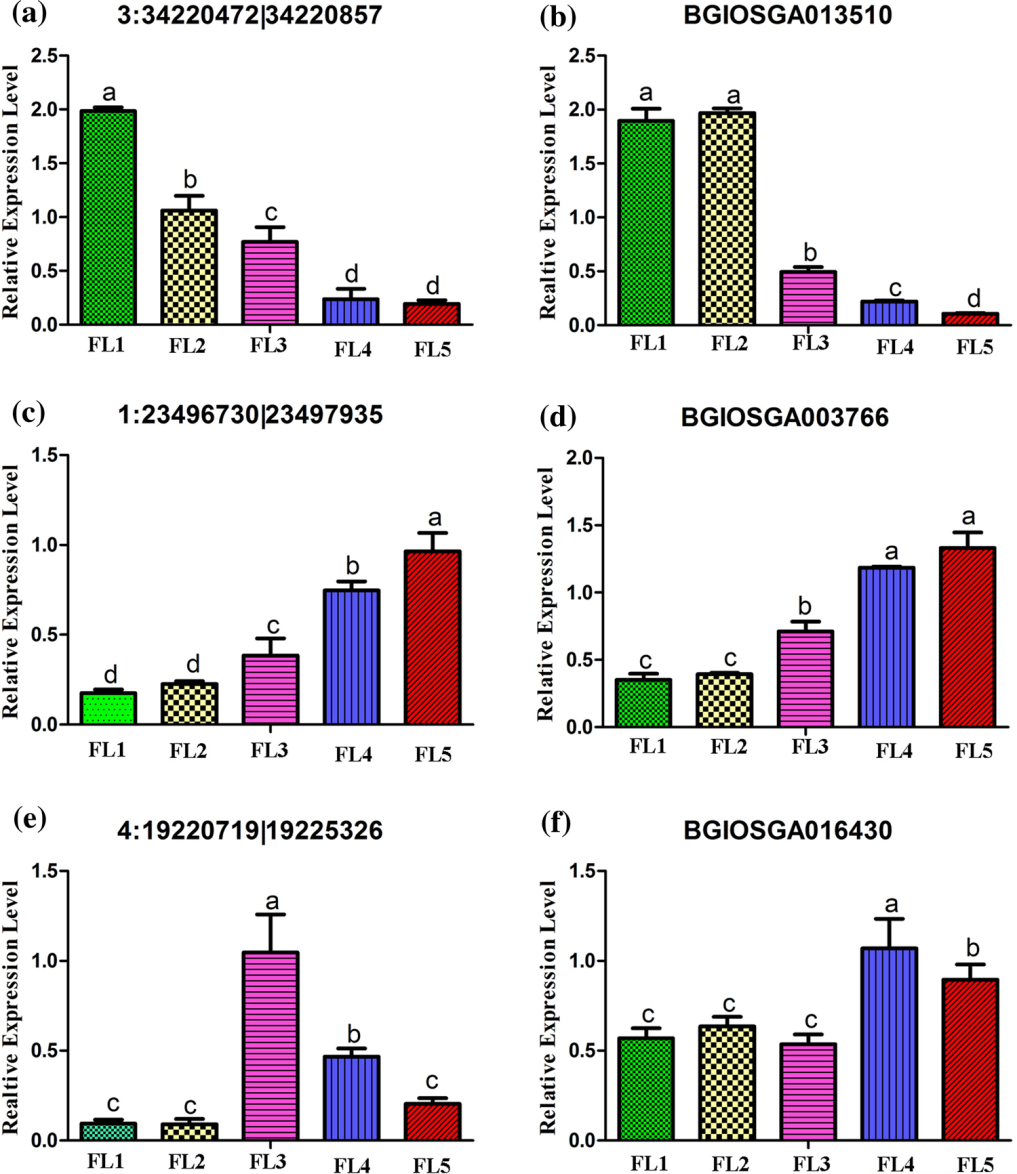
The qRT-PCR analysis of circRNAs and their putative parental genes. **a** Down-regulated circRNA 3:34, 220, 472|34, 220, 857. **b** BGIOSGA013510, the putative parental gene of circRNA 3:34, 220, 472|34, 220, 857. **c** Up-regulated circRNA 1:23, 496, 730|23, 497, 935. **d** BGIOSGA003766, the putative parental gene of circRNA 1:23, 496, 730|23, 497, 935. **e** Turn-point high-expressed circRNA 4:19, 220, 719|19, 225, 326. **f** BGIOSGA016430, the putative parental gene of circRNA 4:19, 220, 719|19, 225, 326. The data are expressed as the mean ± SD of three biological replicates. Different letters indicate values are statistically different based on one-way ANOVA analysis

CircRNAs were reported to regulate parental genes transcription, thus performing biological functions (Zhang et al. 2013). In this study, the ‘MEbrown’ module contained six DE circRNA transcripts which displayed the continuous negative correlation with leaf senescence (Fig. 5b, Table 2). Among these six DE circRNAs, three were predicted and annotated with parental genes (Table 2). The predicted parental gene BGIOSGA013510 (*Os03g0732100*) of the circRNA 3:34,220,472|34,220,857, was identified to be a TALE transcription factor (Table 2). The predicted parental gene of circRNA 7:21,030,120|21,030,979 was a novel gene predicted to perform posttranslational modification (Table 2). And the predicted parental gene BGIOSGA010739 (*Os03g0348200*) of the circRNA 3:14,657,773|14,658,742 was annotated to possess terpene synthase activity (Table 2). The ‘MEblue’ module comprised 18 DE circRNAs which displayed the continuous positive correlation with leaf senescence (Fig. 5b, Table 2), in which nine parental genes were predicted and annotated (Table 2). Interestingly, a predicted parental gene BGIOSGA004735 (*Os01g0830700*) of circRNA 1:38,955,712|38,956,176 was identified to be a leaf senescence-related protein (Table 2). The parental gene BGIOSGA029180 (*Os11g0671000*) of circRNA 8:29,762,968|29,763,202 was annotated to encode auxin-responsive protein (Table 2). The parental gene BGIOSGA003766 (*Os01g0559600*) of circRNA 1:23,496,730|23,497,935 was annotated to possess cysteine-type endopeptidase activity (Table 2). Furthermore, the ‘MEturquoise’ module contained 15 DE circRNA transcripts (Fig. 5b, Table 2). Parental genes of five DE circRNAs were predicted and annotated whereas the other 10 were not (Table 2). For instance, the predicted parental gene BGIOSGA016430 (*Os04g0429850*) of circRNA 4:19,220,719|19,225,326 was annotated to be involved in NAD biosynthesis (Table 2). The parental gene BGIOSGA033357 (*Os10g0536450*) of circRNA 10:19,878,758|19,879,082 was predicted to participate in the oxidation–reduction process (Table 2). In addition, a predicted parental gene BGIOSGA031532 (*Os10g0521700*) of circRNA 10:19,167,135|19,167,568 was annotated to be involved in signal transduction (Table 2). The expression level of down-expressed circRNA 3:34,220,472|34,220,857 and its parental gene BGIOSGA013510 (*Os03g0732100*), and that of up-expressed circRNA 1:23,496,730|23,497,935 and its parental gene BGIOSGA003766 (*Os01g0559600*), and that of turn-point circRNA 4:19,220,719|19,225,326 and its parental gene BGIOSGA016430 (*Os04g0429850*) were verified by qRT-PCR (Fig. 6), and the results indicated that the abundances of circRNAs were positively correlated with the expression of their parental gene. These results implied that the senescence-associated circRNAs possibly performed functions in flag leaf senescence through regulating their parental genes.

**Table 2:** Thirty-nine interested senescence-associated circRNAs clustered in three modules

circRNA ID	circRNA module	Parental gene	RAP-ID	Description
3:34,220,472|34,220,857	Brown	BGIOSGA013510	Os03g0732100	Regulation of transcription, DNA-templated; TALE family
7:21,030,120|21,030,979	Brown	Oryza_sativa_newGene_32874	–	Posttranslational modification
3:14,657,773|14,658,742	Brown	BGIOSGA010739	Os03g0348200	Terpene synthase activity
3:35,636,429|35,642,189	Brown	–	–	–
3:39,797,208|39,848,986	Brown	–	–	–
12:9,093,145|9,130,285	Brown	–	–	–
2:33,014,336|33,015,119	Blue	BGIOSGA005647	Os02g0741500	mRNA export from nucleus
1:43,314,777|43,317,734	Blue	BGIOSGA000285	–	Posttranslational modification
1:38,955,712|38,956,176	Blue	BGIOSGA004735	Os01g0830700	O-acetyltransferase activity, leaf senescence-related protein
8:29,762,968|29,763,202	Blue	BGIOSGA029180	Os11g0671000	Auxin-responsive protein
3:10,405,683|10,408,702	Blue	BGIOSGA010981	Os03g0280000	ABC transporter family protein, putative, expressed
1:23,496,730|23,497,935	Blue	BGIOSGA003766	Os01g0559600	Cysteine-type endopeptidase activity
5:23,221,948|23,223,370	Blue	BGIOSGA017954	Os05g0446800	Arginyl-tRNA–protein transferase
6:30,705,916|30,706,427	Blue	BGIOSGA020637	Os06g0691000	DNA repair protein REV1
7:26,274,523|26,274,796	Blue	BGIOSGA026355	Os07g0669200	Probable GTP-binding protein OBGC1, chloroplastic
1:27,279,428|27,280,791	Blue	–	–	–
2:19,575,625|19,667,206	Blue	–	–	–
4:20,185,567|20,187,969	Blue	–	–	–
5:24,720,286|24,721,772	Blue	–	–	–
5:25,797,150|25,797,312	Blue	–	–	–
6:9,633,683|9,634,101	Blue	–	–	–
8:20,488,115|20,509,065	Blue	–	–	–
8:29,567,424|29,568,252	Blue	–	–	–
8:5,251,329|5,319,040	Blue	–	–	–
8:17,248,521|17,266,134	Turquoise	Oryza_sativa_newGene_35329;Oryza_sativa_newGene_35330	–	Posttranslational modification
4:19,220,719|19,225,326	Turquoise	BGIOSGA016430	Os04g0429850	NAD biosynthetic process
10:19,878,758|19,879,082	Turquoise	BGIOSGA033357	Os10g0536450	Oxidation–reduction process
5:2,405,419|2,405,859	Turquoise	BGIOSGA019146	Os05g0136900	N-acyltransferase activity
10:19,167,135|19,167,568	Turquoise	BGIOSGA031532	Os10g0521700	Signal transduction
1:23,810,695|23,813,009	Turquoise	–	–	–
1:26,469,017|26,477,623	Turquoise	–	–	–
1:31,309,262|31,309,883	Turquoise	–	–	–
1:36,318,548|36,400,367	Turquoise	–	–	–
2:29,835,008|29,836,416	Turquoise	–	–	–
4:15,277,633|15,363,855	Turquoise	–	–	–
4:15,306,141|15,363,867	Turquoise	–	–	–
8:17,332,523|17,383,277	Turquoise	–	–	–
8:17,593,923|17,612,480	Turquoise	–	–	–
9:14,657,454|14,658,026	Turquoise	–	–	–

Note: ‘–’ indicates no corresponding parental gene or RAP-ID

### Putative functions of the interested senescence-associated circRNAs acting as miRNA sponges

**Fig. 7:**
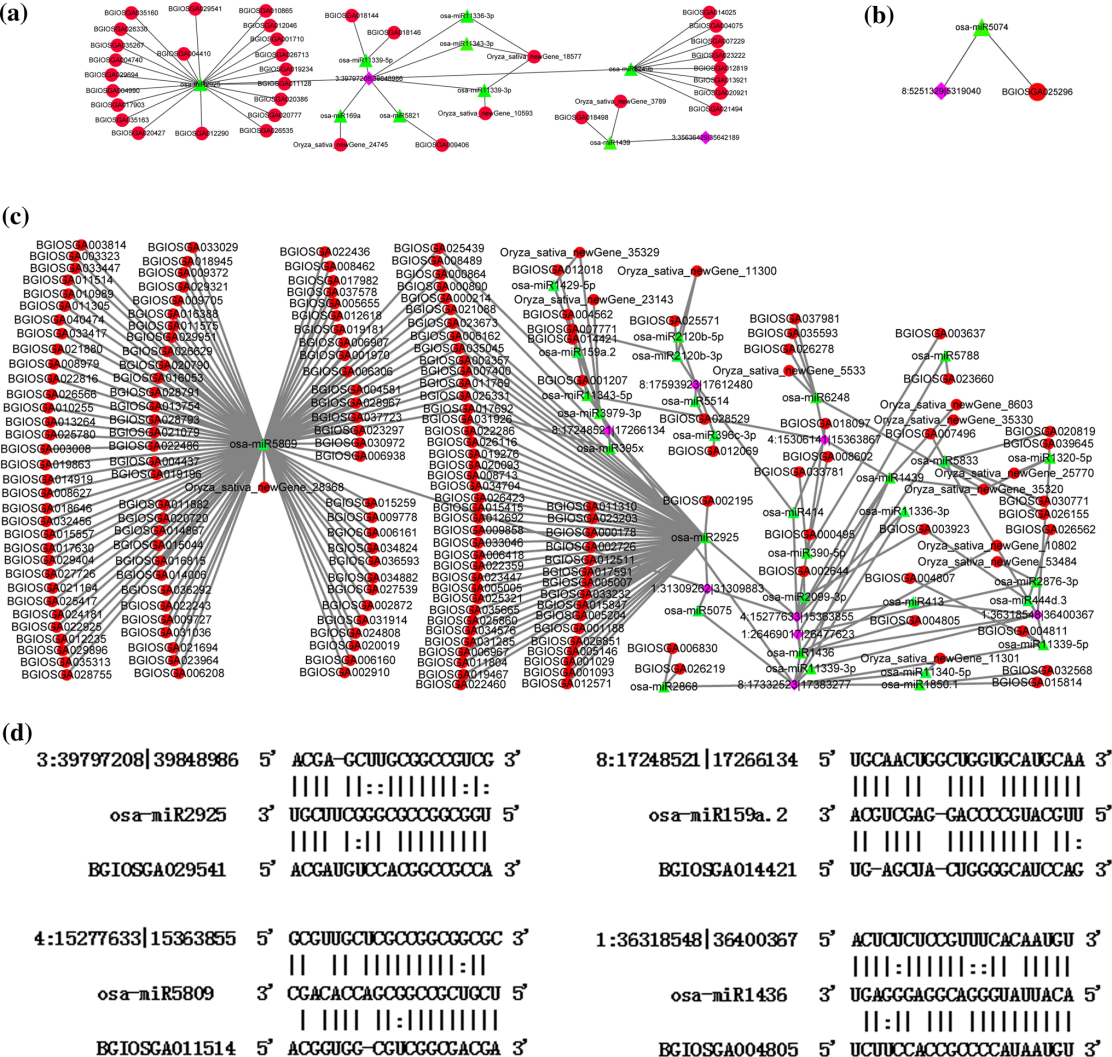
The circRNAs-associated ceRNA networks formed by down-regulated, up-regulated and turn-point high-expressed circRNAs, and their co-expressed mRNAs. **a** The ceRNA network formed by down-regulated circRNAs and their co-expressed mRNAs in ‘MEbrown’ module. **b** The ceRNA network formed by up-regulated circRNAs and their co-expressed mRNAs in ‘MEblue’ module. **c** The ceRNA network formed by turn-point circRNAs and their co-expressed mRNAs in ‘MEturquoise’ module. Diamond, triangle and circle indicated the circRNAs, miRNAs and mRNAs, respectively. **d** The binding sites of circRNA, miRNA and the corresponding mRNA

It has been reported that circRNAs could act as targets of miRNAs, also known as miRNA sponges, to regulate the target genes of corresponding miRNAs by the ceRNA networks (Hansen et al. 2013). In addition, circRNAs were reported to have similar expression patterns in the ceRNA network (Liu et al. 2020). In the present study, three ceRNA networks were constructed using senescence-associated DE circRNAs and DE mRNAs from the ‘MEbrown’, ‘MEblue’ and ‘MEturquoise’ modules, respectively, combining with miRNAs from miRBase (release 22, October 2018). Hence, we first predicted the potential of senescence-associated DE circRNAs to act as targets for miRNAs based on the sequence complementarity. Then, we further identified the miRNAs targeting mRNAs. A total of 11 DE circRNAs were identified as potential of miRNAs sponges, which regulated the expression of target genes through ceRNA networks (Fig. 7a-c). The detailed description is displayed as follows:

For down-regulated DE circRNAs and their co-expressed DE mRNAs in ‘MEbrown’ module, a total of 2 competitive relationship sub-networks were identified, including 2 circRNAs, 9 miRNAs and 37 mRNAs (Fig. 7a). For instance, it was found that circRNA 3:39, 797, 208|39, 848, 986 may sponge osa-miR2925 to regulate 21 target mRNAs (Fig. 7a). For ‘MEblue’ module, the ceRNA network constructed by up-regulated DE circRNAs and their co-expressed DE mRNAs, a circRNA 8:5, 251, 329|5, 319, 040 was predicted to be a possible sponge of miRNA osa-miR5074 to regulate the expression of mRNA BGIOSGA025296 (*Os07g0187900*) (Fig. 7b). Furthermore, using the DE circRNAs and their co-expressed DE mRNAs in the turn-point ‘MEturquoise’ module, the ceRNA network was constructed and identified to contain 8 circRNAs, 25 miRNAs and 193 mRNAs (Fig. 7c). It was found that circRNA 4:15,277,633|15,363,855 may bind with osa-miR5809 to regulate 107 mRNAs. The illustrations of the four circRNAs were provided to display the interaction of the circRNA, miRNA and the corresponding target mRNA (Fig. 7d).

A GO functional enrichment analysis was performed using all these protein-coding genes in ceRNA networks. The results suggested that these mRNAs were involved in many biological processes associated with leaf senescence, including ‘protein phosphorylation (GO:0,006, 468)’, ‘oxidation–reduction process (GO:0,055, 114)’, ‘response to abscisic acid (GO:0,009, 737)’, ‘fatty acid biosynthetic process (GO:0,006, 633)’, ‘signal transduction (GO:0,007, 165)’, ‘membrane lipid metabolic process (GO:0,006, 643)’, ‘cytokinin-activated signaling pathway (GO:0,009, 736)’, ‘lipid catabolic process (GO:0,016, 042)’, and ‘negative regulation of programmed cell death (GO:0,043, 069)’ (Suppl. Table S8). In addition, since many biological processes are reported to be associated with leaf senescence, according to the annotation information for mRNAs, these protein-coding genes in ceRNA networks were further classified into different categories referring to those well-known senescence-related biological processes, including transcription regulation, posttranslational modification (especially protein glycosylation), oxidation–reduction process, hormone signaling pathway, proteolysis and DNA damage repair (Suppl. Table S9). Eventually, a total of 13 target genes were various transcription factors, belonging to NAC, bHLH, HD-ZIP, SBP, ZF-HD, WRKY, MYB and ERF transcription factor families identified by searching on Plant Transcription Factor Database (http://planttfdb.cbi.pku.edu.cn/). Seventeen target genes were annotated to be related with posttranslational modification and another eight were with protein glycosylation. Twenty-two target genes were related with oxidation–reduction process. Eighteen hormone-related genes were involved in the signaling pathways of jasmonic acid, salicylic acid, abscisic acid, ethylene, cytokinin and auxin. Six target genes were associated with proteolysis and three with DNA damage repair. Thus, the senescence-associated circRNAs might play important roles in flag leaf senescence by regulating a large number of mRNAs related to senescence processes through the ceRNA regulatory network.

**Fig. 8:**
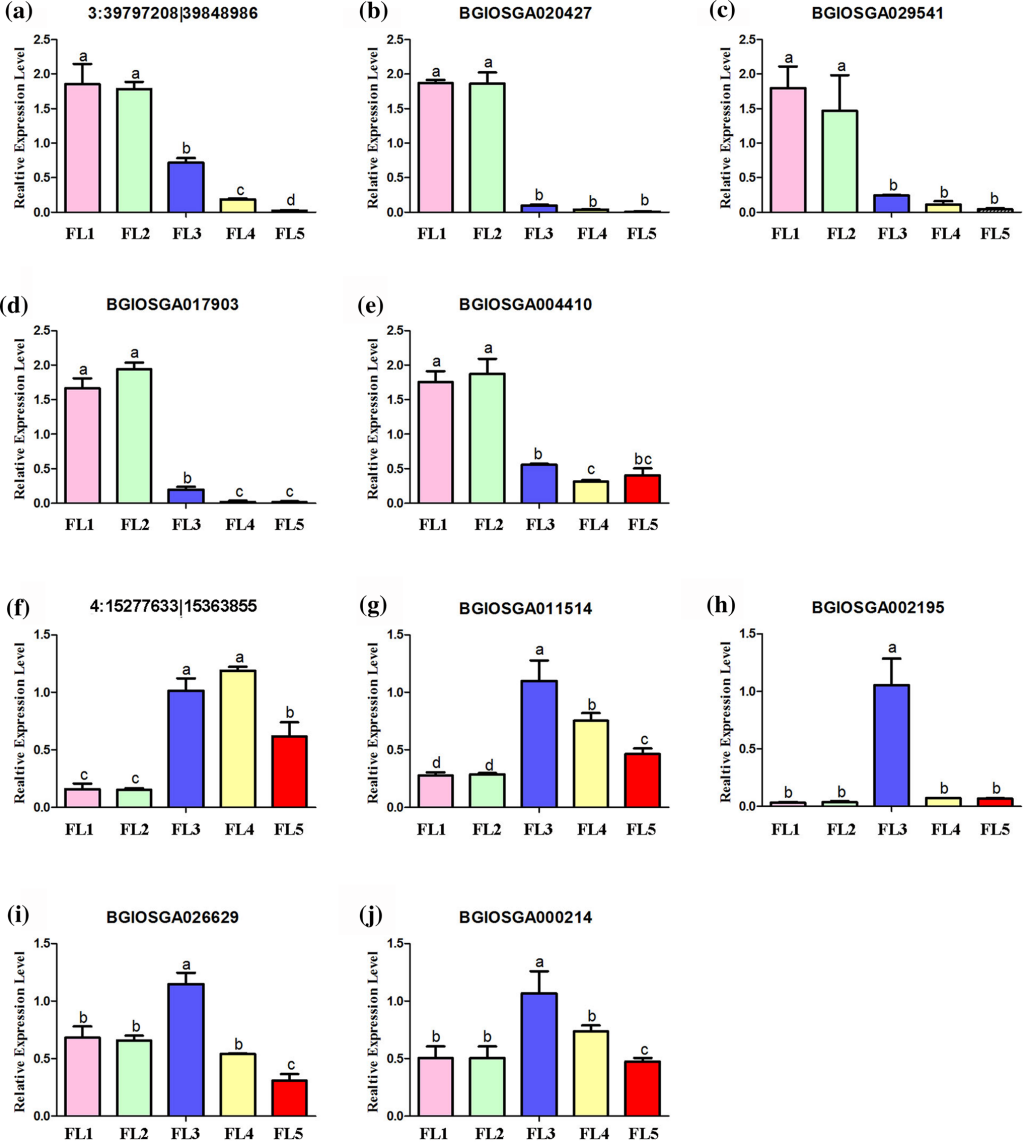
The qRT-PCR validation of DE circRNAs and their co-expressed and possibly regulated mRNAs in ceRNA network. **a** Down-regulated circRNA 3:39, 797, 208|39, 848, 986. **b** mRNA BGIOSGA020427. **c** mRNA BGIOSGA029541. **d** mRNA BGIOSGA017903. **e** mRNA BGIOSGA004410. **f** Turn-point high-expressed circRNA 4:15, 277, 633|15, 363, 855. **g** mRNA BGIOSGA011514. **h** mRNA BGIOSGA002195. **i** mRNA BGIOSGA026629. **j** mRNA BGIOSGA000214. The data are expressed as the mean ± SD of three biological replicates. Different letters indicate values are statistically different based on one-way ANOVA analysis

To validate the regulatory relationship between circRNAs and mRNAs, qRT-PCR experiments were performed. In the ceRNA networks, expression levels of two DE circRNAs and eight mRNAs were confirmed. The results showed that expression levels of the senescence-downregulated circRNAs 3:39, 797, 208|39, 848, 986 and its putatively regulated mRNAs including BGIOSGA020247 (ZF-HD family transcription factor), BGIOSGA029541 (SBP family transcription factor), BGIOSGA004410 (glycosyltransferase), and BGIOSGA017903 (glycosyltransferase) decrease with the flag leaf developing to senescence (Fig. 8a-e), suggesting that circRNA 3:39, 797, 208|39, 848, 986 might regulate the expression of these mRNAs through binding with osa-miR2925. Similarly, the turn-point high-expressed circRNA 4:15, 277, 633|15, 363, 855 might bind with osa-miR5809 to regulate the expression levels of protein-coding genes BGIOSGA011514 (NAC transcription factor gene *ONAC022*), BGIOSGA002195 (cytokinin oxidase 2), BGIOSGA026629 (auxin-responsive gene) and BGIOSGA000214 (probable glucuronosyltransferase), all showing higher expression levels at the turn-point stage (FL3) of the leaf senescence process (Fig. 8f-j). These key circRNAs forming the ceRNA networks may play important functions in leaf senescence.

## Discussion

### Systematic identification and characterization analysis of circRNAs enriched understanding on leaf senescence in rice

CircRNAs are a class of special non-coding RNAs, which have attracted the increasing number of attentions. Accumulating evidences have demonstrated that circRNAs play important functions in various biological processes including stress, growth and development (Barrett and Salzman 2016). With the rapid development of high-throughput sequencing and bioinformatics technology, a vast number of circRNAs have been identified in plants, such as trifoliate orange (Zeng et al. 2018), cucumber (Zhu et al. 2019), wheat (Xu et al. 2019), *Arabidopsis* (Chen et al. 2017b) and maize (Han et al. 2020), revealing their important roles in multiple different biological process. Some plant development related circRNAs were also identified. For example, a total of 3174 circRNAs were characterized during leaf bud to young leaf development in tea (Tong et al. 2018). A total of 2616 circRNAs in sea buckthorn fruit development was identified, of which 252 circRNAs were differentially expressed between three different developmental stages (Zhang et al. 2019). However, to our knowledge, no systematic identification of circRNAs involving in leaf senescence of rice was reported. In this study, a total of 6612 circRNAs were identified by high-throughput sequencing during the developmental process of rice flag leaf from normal to senescence (Suppl. Table S2). The number of circRNAs identified in this study was larger than that in leaf development of tea (558) (Tong et al. 2018), in leaf and root of cucumber under salt stress (2787) (Zhu et al. 2019), in roots, stems, leaves, flowers and siliques of *Arabidopsis* across the lifespan (5861) (Chen et al. 2017b), in maize leaf under different stress treatment (1199) (Han et al. 2020), in wheat leaves in the dehydration stress response (88) (Wang et al. 2017b), in potato stem tissues in response to *Pectobacterium carotovorum* subspecies brasiliense infection (2098) (Zhou et al. 2018), and in tomato during fruit ripening process (705) (Yin et al. 2018), but less than that in fertility transition of rice photo-thermosensitive genic male sterile line (9994) (Wang et al. 2019). The reason for the number difference of identified circRNAs may be attributed to the difference of sample type and number, and most probably to the variety of plant species. The large number of circRNAs identified in this study suggested that circRNAs may contain one of the largest RNA families in rice flag leaf transcription. It may increase the potential influence of non-coding RNAs on cell function and the complex regulation of biological processes (Errichelli et al. 2017). Therefore, examining the important functions of individual circRNAs will be a theme of future research.

Notoriously, circRNAs can be classified into three categories including exonic circRNAs, intronic circRNAs and intergenic circRNAs based on their location on the genome. Among the identified 6612 circRNAs, exonic circRNAs were predominant circRNAs (62.78%) (Fig. 2a). This result is accordance with previous researches, in which the majority of circRNAs were exonic in tea and cucumber (Tong et al. 2018; Zhu et al. 2019). However, a total of 51% and 60.2% of the circRNAs were intergenic circRNAs in kiwifruit and wheat (Wang et al. 2017b, c). In soybean, 48% of total circRNAs were intronic circRNAs, while there are more intronic circRNAs in the root and more exonic circRNAs in the stem (Zhao et al. 2017). A bold speculation was proposed that exonic circRNAs with a low proportion in wheat might result from the huge genome size with comparable fewer available genes, and that introns circRNAs with a higher proportion in soybean might due to the duplicated and huge genome and multiple copy number of genes (Wang et al. 2017b; Zhao et al. 2017). The results may further indicate that the molecular basis of circRNAs biogenesis in plants is quite complicated. Simultaneously, distribution analysis suggested that chromosome 1 produced more circRNAs than others (Fig. 2b). It is worthy noticing that 70% parental genes produce one circRNA, although some parental genes produce multiple circRNAs (Fig. 2c), which is consistent with previous reports in plants (Zhang et al. 2019). Thus, an assumption was obtained that these results may indicate the general features of circRNAs in rice.

An increasing number of studies have reported that the sequence conservation of circRNAs was widespread in various plants. For example, in an conservation analysis for two model plants, *Oryza sativa* and *Arabidopsis thaliana*, more than 300 orthologous parental genes generating circRNAs from a similar position were identified, which may imply that circRNAs in plants have the conservation feature as in animals (Ye et al. 2015). Recently, the result of conservation analysis on soybean circRNAs suggested that 551 parental gene pairs generating exonic circRNAs among *Glycine max*, *Oryza sativa* and *Arabidopsis* were orthologs (Zhao et al. 2017). In this present study, a total of 1407 circRNAs in *Oryza sativa* had sequence conservation compared with those of other plant species in the PlantcircBase database (Suppl. Table S4). Generally, the sequence conservation of circRNAs demonstrated that these circRNAs may have similar potential biological functions, and the functions of these conserved circRNAs in the plants need further investigation and verification.

### Senescence-associated circRNAs involved in flag leaf senescence of rice probably by regulating their parental genes

Functional studies on circRNAs are very limited, particularly in various plants. Here, a WGCNA method was employed to investigate the co-expression network of 113 DE circRNAs and 9412 DE protein-coding mRNAs and further inferred the potential biological functions of circRNAs. Interestingly, three out of seven modules were considered to be highly associated with flag leaf senescence (Fig. 5). The DE circRNAs clustered in these three modules (‘MEbrown’, ‘MEblue’ and ‘MEturquoise’) were considered as the senescence-associated circRNAs (Table 2), and the functions of their parental genes were further discussed.

Increasing evidence has suggested that TALE transcription factor could regulate genes involved in growth and development (Sentoku et al. 1999). In this study, the parental gene BGIOSGA013510 (*Os03g0732100*) of a down-regulated circRNA 3:34, 220, 472|34, 220, 857 was identified to be a TALE transcription factor (Table 2), and qRT-PCR results showed that the abundance of circRNA 3:34, 220, 472|34, 220, 857 was positively correlated with that of mRNA BGIOSGA013510 (Fig. 6a, b), which indicated this circRNA may play important roles in leaf senescence by regulating transcription factor. After gene transcription, mRNAs will be exported from nucleus to cytoplasm for protein translation. And the parental gene of circRNA 2:33, 014, 336|33, 015, 119 is annotated to perform this function (Table 2). Many proteins need posttranslational modification to play their roles. Protein glycosylation, phosphorylation, S-nitrosylation and SUMOylation were found to play important roles in the senescence process of animals and plants (Xiao et al. 2015; Princz and Tavernarakis 2017; Rizza et al. 2018; Ke et al. 2019). Interestingly, in this study, the parental genes of a down-regulated circRNA 7:21, 030, 120|21, 030, 979, an up-regulated circRNA 1:43, 314, 777|43, 317, 734 and a turn-point high-expressed circRNA 8:17, 248, 521|17, 266, 134 were all annotated to perform the function of posttranslational modification (Table 2). It is well known that oxidation–reduction processes are related to leaf senescence. A number of terpene synthases from a range of plant species have been characterized, and terpene synthase gene *OsTPS20* was found to be induced by oxidative stress (Lee et al. 2015). Accordingly, the parental gene BGIOSGA010739 (*Os03g0348200*) of a down-regulated circRNA 3:14, 657, 773|14, 658, 742 was annotated to possess terpene synthase activity (Table 2), which may play role in leaf senescence through participating in reactive oxygen signaling pathway. Furthermore, NAD plays critical roles in cellular redox reactions to prevent cell death, and functional validation of a gene *OsNaPRT1* encoding nicotinate phosphoribosyltransferase has suggested that the deficiency in the NAD salvage pathway trigger premature leaf senescence through reactive oxygen accumulation and transcriptional activation of senescence-related genes (Wu et al. 2016). In this study, the parental gene BGIOSGA016430 (*Os04g0429850*) of a turn-point circRNA 4:19,220,719|19,225,326 was annotated to be a nicotinate phosphoribosyltransferase (Table 2), and qRT-PCR results showed that the abundance of circRNA 4:19,220,719|19,225,326 was positively correlated with that of mRNA BGIOSGA016430 (Fig. 6e, f). Thus, a bold speculation is proposed that the turn-point circRNA 4:19, 220, 719|19, 225, 326 may play key roles in leaf senescence by involving in NAD biosynthesis pathway to mediate cellular redox status. In addition, a parental gene BGIOSGA033357 (*Os10g0536450*) of turn-point circRNA 10:19, 878, 758|19, 879, 082 was also found to involve in the oxidation–reduction process, which may imply its corresponding role in leaf senescence (Table 2).

So far, a vast number of senescence-associated genes were identified to play roles in leaf senescence (Shim et al. 2019). Interestingly, a parental gene BGIOSGA004735 (*Os01g0830700*) of an up-regulated circRNA 1:38,955,712|38,956,176 in this study was annotated to be a leaf senescence-associated protein with O-acetyltransferase activity on the Rice Genome Annotation Project (http://rice.plantbiology.msu.edu/), which may indicate its roles in leaf senescence (Table 2). N-acyltransferases are also associated with senescence. N-acetyltransferase 10 was found to be involved in enhancing healthspan in a mouse model of human accelerated aging syndrome (Balmus et al. 2018). Overexpression of rice serotonin N-acetyltransferase 1 in transgenic rice plants confers resistance to cadmium and senescence and increases grain yield (Lee and Back 2017). In our results, the parental gene BGIOSGA019146 (*Os05g0136900*) of a turn-point high-expressed circRNA 5:2, 405, 419|2, 405, 859 was annotated with N-acyltransferase activity (Table 2). Hormones and their signal transduction pathways are well-known to be involved in leaf senescence (Ellis et al. 2005; Lim et al. 2007; Zhang and Zhou 2013). And ABC transporter proteins were reported to be involved in abscisic acid transport and response (Kuromori et al. 2010), and to participate in senescence-like process in flag leaf (Krattinger et al. 2009). The parental genes of two up-regulated circRNAs 8:29,762,968|29,763,202 and 3:10, 405, 683|10, 408, 702, and one turn-point high-expressed circRNA 10:19, 167, 135|19, 167, 568 were annotated to encode an auxin-responsive protein, an ABC transporter family protein and a signal transduction protein, respectively (Table 2). In addition, senescence-associated proteolysis in plants is a complex and controlled process, which is essential for mobilization of nutrients from old or stressed tissues, mainly leaves, to growing or sink organs (Diaz-Mendoza et al. 2016). GO enrichment analysis on parental genes of DE circRNAs suggested the most significant terms were enriched in ‘proteolysis (GO: 0,006, 508)’ (Fig. 4). The parental gene BGIOSGA003766 (*Os01g0559600*) of up-regulated circRNA 1:23, 496, 730|23, 497, 935 was annotated to possess cysteine-type endopeptidase activity (Table 2), and qRT-PCR results showed that the abundance of circRNA 1:23,496,730|23,497,935 was positively correlated with that of mRNA BGIOSGA003766 (Fig. 6c, d), implying its role in proteolysis. It has been reported that a delayed leaf senescence mutant in *Arabidopsis* is defective in arginyl-tRNA: protein arginyltransferase, a component of the N-end rule proteolytic pathway (Yoshida et al. 2002). The parental gene BGIOSGA017954 (*Os05g0446800*) of up-regulated circRNA 5:23, 221, 948|23, 223, 370 was annotated to be an arginyl-tRNA-protein transferase (Table 2). In *Arabidopsis*, DNA double-strand break can trigger leaf senescence through epigenetic control of senescence-associated genes (Li et al. 2020). The parental gene BGIOSGA020637 (*Os06g0691000*) of up-regulated circRNA 6:30,705,916|30,706,427 was annotated to encode DNA repair protein REV1, which may play role in leaf senescence through involving in DNA repair pathway (Table 2). In addition, leaf senescence is characterized by the gradual degradation of chloroplast. It has been reported that the gene *OBGC1* functioned primarily in plastid ribosome biogenesis during chloroplast development (Bang et al. 2012). In this study, the parental gene BGIOSGA026355 (*Os07g0669200*), encoding a probable GTP-binding protein OBGC1, was regulated by up-regulated circRNA 7:26, 274, 523|26, 274, 796 (Table 2).

**Fig. 9:**
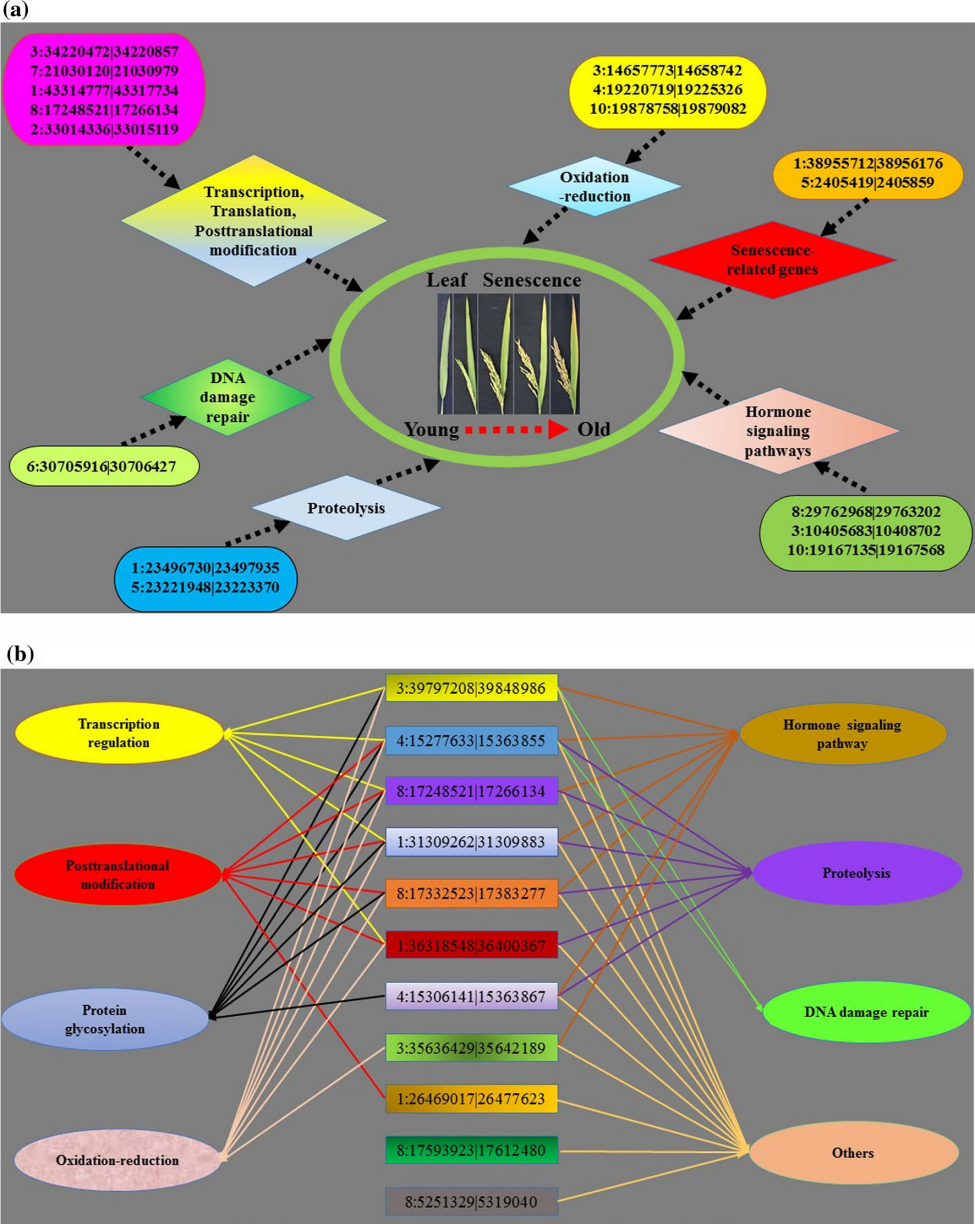
The network models of senescence-associated circRNAs involved in leaf senescence. **a** The model of circRNAs by regulating their parental genes to participate in senescence-related biological processes. **b** The model of circRNAs by regulating mRNAs through ceRNA network to participate in senescence-related biological processes

Therefore, the senescence-associated DE circRNAs identified in this study possibly play roles in leaf senescence by regulating their protein-coding parental genes, which all showed close connection with the well-studied senescence-associated processes, mainly including the processes of transcription, translation, and posttranslational modification, oxidation–reduction process, involvement of senescence-associated genes, hormone signaling pathway, proteolysis, and DNA damage repair (Fig. 9a). However, the functions of these circRNAs need to be further experimentally confirmed by transgenic technology.

### Senescence-associated circRNAs involved in flag leaf senescence of rice possibly through ceRNA regulatory networks

Increasing evidences in eukaryotic species have demonstrated that circRNAs could act as potential miRNA sponges, which sequester miRNAs away from its target mRNAs through circRNAs–miRNAs–mRNAs regulatory networks. For example, a human circRNA circHIPK3 harboring 18 potential binding sites of 9 miRNAs in human cells (Zheng et al. 2016). In the present study, three ceRNA networks were constructed, from which, some key circRNAs acting as miRNA sponges, were found to probably regulate the expression of a large number of mRNAs to participate in flag leaf senescence (Fig. 7). And the possibility of involvement of these mRNAs in leaf senescence was discussed below.

It is well known that transcription factors play vital roles in leaf senescence. Accordingly, in this study, a total of 13 target genes in the ceRNA network were identified to be various transcription factors (Suppl. Table S9). In rice, overexpression of a stress-responsive NAC-type transcription factor *ONAC022* could improve drought and salt tolerance through modulating an ABA-mediated pathway, further showed delayed and less leaf rolling compared with the wild-type plants (Hong et al. 2016). Interestingly, mRNA BGIOSGA011514 (*Os03g0133000*) was identified to be transcription factor *ONAC022* and possibly regulated by circRNA 4:15, 277, 633|15, 363, 855 (Suppl. Table S9). In addition, the HD-ZIP gene family plays important roles in plant growth and development. It has been reported that overexpression of the HD-ZIP gene *OsHox32* could result in pleiotropic effects on plant-type architecture and leaf development in rice (Li et al. 2016). Another HD-ZIP family gene *OsHOX33*, its knockdown could accelerate leaf senescence (Luan et al. 2013). Accordingly, a gene BGIOSGA026851 (*Os08g0465000*) in our ceRNA network was identified to be a HD-ZIP member *OsHOX27*, which may possess the similar functions in leaf senescence. This transcription factor gene is predicted to be regulated by three circRNAs 1:31, 309, 262|31, 309, 883, 4:15, 277, 633|15, 363, 855, and 8:17, 248, 521|17, 266, 134 (Suppl. Table S9).

Considering the important roles of posttranslational modification in senescence. In the ceRNA networks, it was found that 17 mRNAs were annotated to perform posttranslational modification (Suppl. Table S9). Most importantly, increasing evidences have suggested that protein glycosylation play important roles in cell death or senescence. For example, rice *premature leaf senescence 2* (*PLS2*), encoding a glycosyltransferase, is involved in leaf senescence (Wang et al. 2018a). The rice *OsDGL1*, encoding a dolichyl-diphosphooligosaccharide-protein glycosyltransferase 48 kDa subunit precursor, is involved in *N*-glycosylation and cell death in the root (Qin et al. 2013). In our previous study, the UDP-*N*-acetylglucosaminepyrophosphorylase 1 (*UAP1*) was successfully identified to catalyze the biosynthesis of UDP-*N*-acetylglucosamine, which is one of key substrates for *N*-glycan, and function inactivation of *UAP1* induced early leaf senescence and defense responses in rice (Wang et al. 2015). In addition, a large number of *N*-glycoproteins involving in senescence-associated biological processes were found in the senescent flag leaf of rice (Huang et al. 2019). Interestingly, in ceRNA network, another eight target genes were predicted to be involved in glycosylation (Suppl. Table S9). These results imply that senescence-associated circRNAs may play roles in leaf senescence by involving in posttranslational modification, especially protein glycosylation.

The generation of reactive oxygen species is one of the earliest responses of plant cells under abiotic stresses and senescence (Jajic et al. 2015). Thus, oxidation–reduction process is highly considered to be related with leaf senescence. The GO functional enrichment analysis of mRNAs in ceRNA network also showed that ‘oxidation–reduction process (GO: 0, 055, 114)’ was the top second enriched terms (Suppl. Table S8). Optimal levels of reactive oxygen species are required for programed cell death. The plant NADPH oxidase genes known as respiratory burst oxidase homolog (RBOH) genes play a role in the generation of reactive oxygen species (Zheng et al. 2019). In our results, BGIOSGA017692 (*Os05g0528000*) is identified as the *OsRBOHc* gene, and two circRNAs 4:15, 277, 633|15, 363, 855 and 8:17, 248, 521|17, 266, 134 possibly participated in flag leaf senescence by regulating this gene (Suppl. Table S9). In addition, a novel gene, *OZONE-RESPONSIVE APOPLASTIC PROTEIN1* (*OsORAP1*), enhances cell death in ozone stress in rice by influencing the production of reactive oxygen species (Ueda et al. 2015). In this study, BGIOSGA029896 (*Os09g0365900*) is the *OsORAP1* gene, and it might be regulated by circRNA 4:15, 277, 633|15, 363, 855 in the ceRNA network (Suppl. Table S9). At last, a total of 22 protein-coding genes regulated by seven circRNAs in the ceRNA network were predicted to be involved in oxidation–reduction process (Suppl. Table S9).

Phytohormones, including jasmonic acid, salicylic acid, abscisic acid, ethylene, cytokinin and auxin, were considered to play roles in various biological process including plant growth, development and leaf senescence (Lim et al. 2007). One of the mechanisms that controlling the endogenous level of cytokinins was mediated by the enzyme cytokinin oxidase, which irreversibly degraded cytokinins in various plants (Galuszka et al. 2001). It has been reported that down-regulated expression of cytokinin oxidase 2 increases tiller number, delays senescence and further improves rice yield (Yeh et al. 2015). In the present study, the target gene BGIOSGA002195 (*Os01g0197700*) was identified to be the cytokinin oxidase 2, and it was predicted to be regulated by three circRNAs 1:31, 309, 262|31, 309, 883, 4:15, 277,633|15, 363, 855, and 8:17, 248, 521|17, 266, 134 (Suppl. Table S9). Moreover, early studies indicate that auxins have a role in the suppression of leaf senescence (Lim et al. 2007). Transgenic rice plants overexpressing the *SAUR39* gene, a negative regulator of auxin synthesis and transport, had lower auxin level, lower chlorophyll content, and faster leaf senescence compared with wild-type plants at the vegetative stage (Kant et al. 2009). The target gene BGIOSGA026629 (*Os08g0524200* or *LOC_Os08g41280*) is a leaf senescence-associated gene encoding an auxin-responsive protein in rice (Liu et al. 2008), and this gene was found to be possibly regulated by circRNA 4:15, 277, 633|15, 363, 855 (Suppl. Table S9). Importantly, a total of 18 target mRNAs in the ceRNA regulatory network were identified to be hormone-related genes (Suppl. Table S9), referring to the signaling pathway of jasmonic acid, salicylic acid, abscisic acid, ethylene, cytokinin and auxin, respectively. The qRT-PCR results showed that the abundance of circRNA 4:15, 277, 633|15, 363, 855 was positively correlated with that of mRNAs BGIOSGA011514, BGIOSGA002195 and BGIOSGA026629 (Fig. 8). Thus, these results indicated the vital roles of circRNAs in leaf senescence by regulating hormone signaling pathways.

It has been reported that plant senescence and proteolysis were considered to be two processes with one destiny (Diaz-Mendoza et al. 2016). In this study, a total of six target mRNAs regulated by six circRNAs in the ceRNA network were considered to be related to proteolysis biological process (Suppl. Table S9). Two circRNAs were predicted to regulate three mRNAs involving in DNA damage repair (Suppl. Table S9).

In summary, the senescence-associated DE circRNAs identified in this study possibly play key roles in leaf senescence through ceRNA network to mediate transcription regulation, posttranslational modification (especially protein glycosylation), oxidation—reduction process, hormone signaling pathway, proteolysis and DNA damage repair (Fig. 9b).

## ***Author contribution statement***

XPH drafted the manuscript; HYZ provided the bioinformatics analysis; QW, RG and XZL performed the paddy field management and carried out the qRT-PCR experiments; WGK, HYS and JLL provided assistance in experiments and data analysis. YJH participated in the experimental design; ZHW provided the overall guidance and the manuscript revision. All authors read and approved the final manuscript.

## Supplementary Information

Below is the link to the electronic supplementary material.

Supplementary file1 (JPG 1259 KB)

Supplementary file2 (XLSX 17 KB)

Supplementary file3 (XLSX 966 KB)

Supplementary file4 (XLSX 2837 KB)

Supplementary file5 (XLSX 850 KB)

Supplementary file6 (XLSX 41 KB)

Supplementary file7 (XLSX 18 KB)

Supplementary file8 (XLSX 3048 KB)

Supplementary file9 (XLSX 22 KB)

Supplementary file10 (XLSX 64 KB)

## Data Availability

The datasets generated during this study are available in the Genome Sequence Archive at the Beijing Institute of Genomics (BIG) Data Center, Chinese Academy of Sciences, under accession number CRA003505 (http://bigd.big.ac.cn/gsa/s/YB3P26qq).

## Abbreviations

ceRNA: Competing endogenous RNA
circRNAs: Circular RNAs
DE: Differentially expressed
GO: Gene Ontology
KEGG: Kyoto Encyclopedia of genes and genomes
WGCNA: Weighted gene co-expression network analysis
