# Supplementary figures and images for: Systematic identification and characterization of circular RNAs involved in flag leaf senescence of rice
Source: Planta. 2021 Jan 7;253(2):26. doi: 10.1007/s00425-020-03544-6 (PMC7790769; doi:10.1007/s00425-020-03544-6)

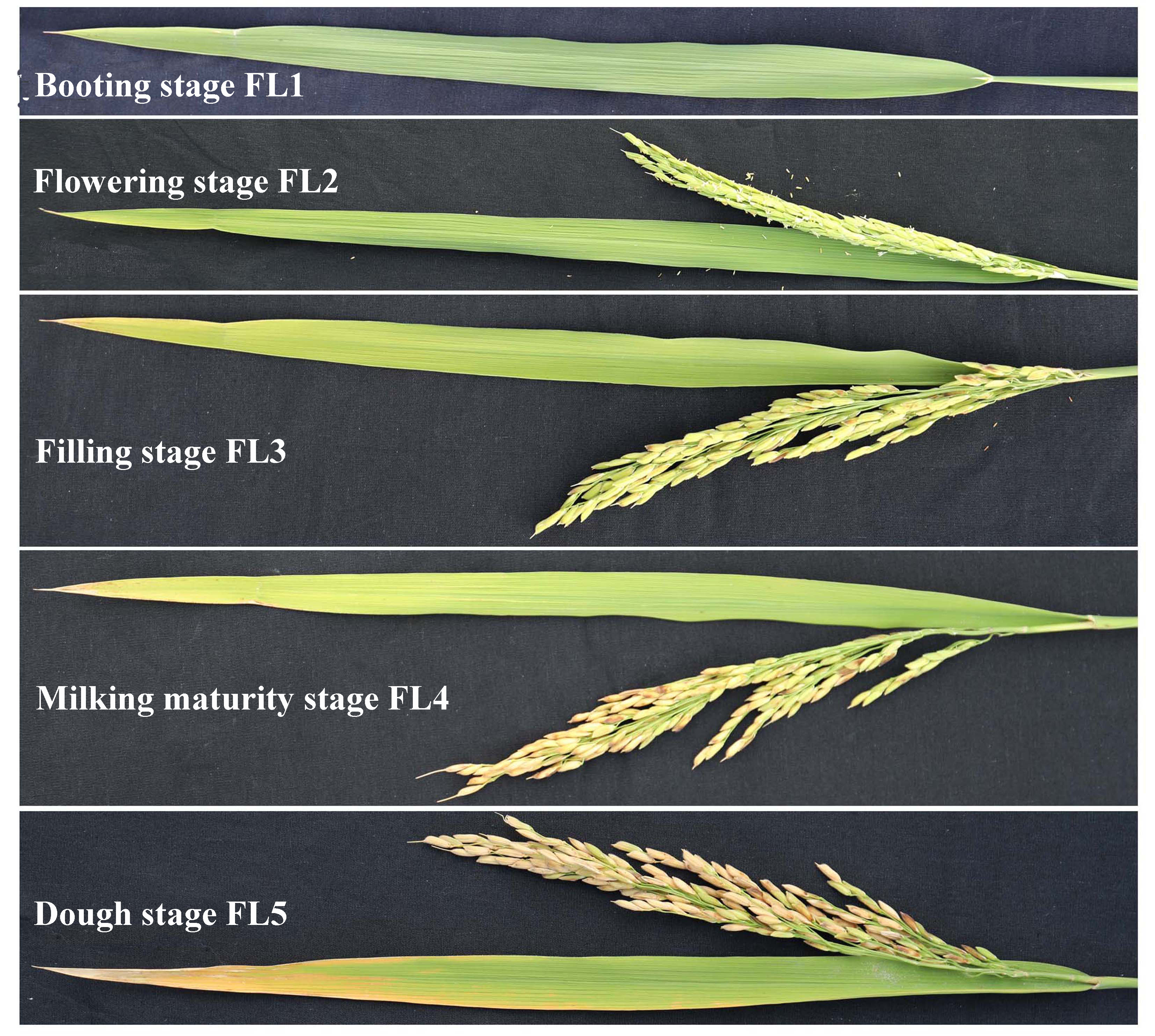

Supplement: Supplementary file 1 — Supplementary file1 (JPG 1259 KB) [file 425_2020_3544_MOESM1_ESM.jpg]
